# Effect of biochar and bioorganic fertilizer on the microbial diversity in the rhizosphere soil of *Sesbania cannabina* in saline-alkaline soil

**DOI:** 10.3389/fmicb.2023.1190716

**Published:** 2023-06-29

**Authors:** Yin-yu Gu, Xiao-yan Liang, Hai-yang Zhang, Rao Fu, Meng Li, Chuan-jie Chen

**Affiliations:** Shandong Institute of Sericulture, Shandong Academy of Agricultural Sciences, Yantai, China

**Keywords:** effect, biochar, bioorganic fertilizer, microbial diversity, saline-alkali rhizosphere soil

## Abstract

**Introduction:**

Biochar and bioorganic fertilizer (BOF) application in agriculture has garnered increasing interest recently. However, the effects of biochar and BOF on rhizosphere soil microecology, especially in a region with saline-alkaline soil, remain largely unexplored.

**Methods:**

In this study, we performed Illumina-based 16S rRNA sequencing to investigate the effects of biochar with or without BOF addition, as well as at different addition rates and particles sizes, on the microecology of saline-alkaline rhizosphere soil.

**Results:**

In the field experiment, biochar and BOF application altered the rhizosphere soil microecology. Actinobacteriota, Proteobacteria, and Chloroflexi accounted for >60% of the total bacterial population in each treatment. In the different treatments, Actinobacteria and Alphaproteobacteria were the predominant classes; Micromonosporales and Vicinamibacterales were the dominant orders; norank_f__Geminicoccaceae and Micromonosporaceae were the most abundant families; and Micromonospora and norank_f_Geminicoccaceae were the predominant genera. Application of biochar with or without BOF decreased soil electrical conductivity (EC) by 7% -11.58% only at the depth of 10 cm below the surface, again, soil EC can be significantly reduced by an average of 4% at 10 cm depth soil after planting Sesbania cannabina. Soil organic carbon, organic matter, available potassium, and available phosphorus contents had significant effects on the soil bacterial community structure.

**Conclusion:**

Co-application of biochar and BOF resulted in the greatest improvement of rhizosphere soil microecology, either by promoting plant growth or improving the nutrition and physicochemical properties of soil, followed by BOF alone and biochar alone. Additionally, higher application rate of biochar was better than lower application rate, and fine biochar had a stronger effect than coarse biochar. These results provide guidance for the development of new saline-alkaline soil remediation strategies.

## 1. Introduction

Soil salinization is a common abiotic stress that reduces the water-extraction capacity of roots and disrupts plant metabolism, in turn affecting crop growth and yield in agroecosystems on a global scale (Yang et al., 2021). Moreover, high salinity not only affects resources, environment, and ecology but also hampers sustainable socioeconomic development. Therefore, the salinization of soils has become a global concern, and many countries are actively researching the underlying causes and developing measures to address this problem. Yellow River Delta, located in the Shandong Province of China, is one of the few large river deltas in the world, with 620,000 hm^2^ of saline-alkaline land that has not yet been extensively developed (Zheng et al., 2020). Since this region is considered a unique land-based resource in Shandong, its restoration aimed at replenishing arable land was undertaken, leading to the improvement of a large portion of the saline land and its utilization for agricultural production, supporting both economic and social development. Several studies have examined the physical, chemical, biological, and engineering improvements resulting from the restoration of saline land (Saleh and Madany, 2015; Hidri et al., 2016; Rajput et al., 2016; Zhao et al., 2018). Among them, eco-friendly amendments and bioremediation are highly praised for their sustainability, such as poultry manure, spent mushroom substrate, plant growth-promoting bacteria (PGPB) or along with CRISPR and nanotechnological approaches significantly reduced adversity stress by increasing the availability of nutrients, phytostimulation activities in plant, and enzymatic activities of the rhizospheric saline soil (Upadhyay and Chauhan, 2022; Upadhyay et al., 2022a; Chauhan and Upadhyay, 2023). Biochar and bioorganic fertilizers (BOFs) have been identified as effective ecological measures for improving the saline-alkaline land (Lu et al., 2020).

Biochar is a carbon-rich byproduct derived from the burning of organic material such as wood, manure, or leaves, under minimal oxygen concentrations through a process called pyrolysis [International Biochar Initiative (IBI), 2015]. Because biochar exhibits good physical and chemical properties, has several ecological advantages, and is abundant and renewable, its application has drawn attention for agricultural production and environmental resource development (Hunt et al., 2010; Liu et al., 2021). Decades of research shows that biochar contains 40%–75% carbon and is characterized by a large specific surface area, rich pore structure, good aeration, low mass and density, and strong adsorption capacity (Abukari and Duwiejuah, 2019; Yang et al., 2021). Therefore, biochar has been widely used as a soil amendment to improve soil fertility (Yuan et al., 2011). Biochar has also been used in research studies for the remediation of saline soils, increasing plant biomass (Drake et al., 2016), improving soil physicochemical properties (Fei et al., 2019; Duan et al., 2021), enhancing soil nutrient levels (Agbna et al., 2017; Xiao and Meng, 2020), and changing soil microbial structure (Qiao et al., 2020). Overall, the conversion of biowaste into biochar, as a soil amendment, has agronomic and environmental benefits and has gained considerable research interest (Qiao et al., 2020).

BOFs are processed from organic substrates, and then mixed with beneficial living microbes (Jena et al., 2020). Possessing the advantages of both traditional organic and microbial fertilizers, BOFs effectively promote fertilizer use efficiency (Yu et al., 2019), reduce chemical fertilizer application (Jena et al., 2020), improve soil quality (Moraditochaee et al., 2014), increase crop yield and quality (Li et al., 2018), and enhance crop disease and stress resistance (Tao et al., 2020). BOFs function by inducing the rapid reproduction of functional bacteria that optimize soil microbial population structure (Jia et al., 2010), enhance soil enzyme activity (Zhao et al., 2016), activate soil nutrients (Gao et al., 2020), improve root vitality (Bhardwaj et al., 2014), and promote root absorption and the use of nutrient elements (Jena et al., 2020). Thus, BOFs are critical for the development of green organic agriculture, and have become a focus of related disciplines worldwide (Nisar et al., 2021). In addition, BOFs have been shown to improve the structural properties and microecological environment of saline-alkaline land (Hafez et al., 2021a) and to enhance the nutrient buffering capacity of soil to prevent salt build-up (Lu et al., 2020), thus serving as an effective tool for the remediation of saline soils (Hafez et al., 2021b). However, few studies have evaluated the combined use of BOFs and biochar for the reclamation of saline-alkaline land.

Salt stress affects all the major processes of plant such as germination, growth, photosynthetic pigments and photosynthesis, water relation, nutrient imbalance, oxidative stress, and yield by causing osmotic and ionic stress (Chauhan P. K. et al., 2022). Some of the plants have the ability to grow under salinity due to the presence of different mechanisms in them for salt tolerance, such plants are known as salt resisting plants, salt tolerating plants or halophytes (Aslam, 2011). *Sesbania cannabina* is highly adaptable to adverse environmental conditions, such as salinity, drought, and waterlogging (Ren et al., 2016). Therefore, being a pioneer plant that decreases soil salt content (Li et al., 2016), there are several studies involved in the excellent performance of *S. cannabina* in soil fertility improvement and salinity reduction (Ren et al., 2018; Zheng et al., 2018). In this study, we used *S. cannabina* to examine the effect of biochar and BOF on plant growth, soil physicochemical properties, and soil bacterial community structure in saline-alkaline soil and to explore the feasibility of using these components for soil improvement. We hypothesize that the combined application of biochar with BOF can more significantly improve soil nutrients levels within salt-alkali soil than the single application of biochar or BOF and that the combined application can significantly impact the soil microbial community compositions. Therefore, this study aims to (i) evaluate the effectiveness of combining biochar with BOF of different particle sizes or amounts in improving saline alkali soil. (ii) Determine the characteristics of microbial community diversity changes after different treatments.

## 2. Materials and methods

### 2.1. Biochar and BOF

BOF was produced by Yangfeng Agricultural Technology Co., Ltd. (Weifang, China), with mushroom residue, humic acid, soybean meal and corn residue as the main substrates, along with supplements including three *Bacillus* species (*B. subtilis, B. licheniformis*, and *B. mucilaginous*), with effective bacteria ≥ 500 million/g, OM ≥ 60%, and nitrogen, phosphorus, and potassium content ≥6%–8%. Biochar was produced by Taiyu Bioengineering Co., Ltd. (Qixia, China), using pyrolytic reactor to char the apple stems under 450°C for 24 h. The sample was then milled and passed through 10, 30, and 60 mesh sieves, obtaining samples with pH of 7.49, 7.36, and 7.45, respectively, and electrical conductivity (EC) of 0.357, 0.355, and 0.349 mS/cm, respectively. The larger meshed samples contained the smaller mesh sample.

### 2.2. Field experiments

Field experiments were conducted at the Institute of Modern Agriculture on the Yellow River Delta, Shandong Academy of Agricultural Sciences (118.37°N, 37.17°E), Dongying, China. The experimental plot used for this experiment is a saline-alkaline wasteland with silty clay soil (Zheng et al., 2018). Except CB1_BOF and FB1_BOF, which were applied at the rate of 100 t/ha, other biochar and BOF treatments were applied at rates of 150 t/ha, such as CK (control; neither biochar nor BOF), CB (10 mesh biochar), FB (30 mesh biochar), BOF, and CB1_BOF (10 mesh biochar + BOF), CB2_BOF (10 mesh biochar + BOF), FB1_BOF (30 mesh biochar + BOF), FB2_BOF (30 mesh biochar + BOF). The biochar and BOF were scattered over the soil surface and tilled to a depth of approximately 0–20 cm by raking in November, the field treatment was performed in triplicate by a random block design, with plot in sizes of 4 m × 8 m. Soil was sampled from each plot in March before planting *S. cannabina*. The *S. cannabina* were sowed in the field in April, and no field management practices were performed, except watering. Plant height was measured in August, and rhizosphere soil was collected in June and August. The plant root system was shaken to remove excess soil, and only the tightly adhering soil was used for analysis. The rhizosphere soils of three plants were sampled from a depth of 0–20 cm in each treatment, and the three soils samples per treatment were pooled together, mixed thoroughly, and cleaned to remove impurities. Then, each pooled sample was divided into two sections: one of that was immediately packaged with dry ice after liquid nitrogen quick freezing, and sent to MajorBio for high-throughput sequencing, while the other part was air-dried, homogenized, and sieve through a sieve with an aperture of less than 2 mm to remove any remaining impurities, and used for the measurement of soil physicochemical properties. Biological replicate was conducted in triplicate.

### 2.3. Analysis of soil physicochemical properties

Soil pH was determined using a Magnetic Multi-parameter Water Quality Analyzer (DZS-708; Shanghai Lei). The volumetric weight (VW) of soil samples was determined by the ring knife method (Li et al., 2019). The electrical conductivity (EC) of soil was measured at a depth of 10 cm using the FieldScout EC450 meter. The detection methods of the other nutrients were shown in Supplementary Table S1.

### 2.4. Soil microbial community analysis

Genomic DNA was extracted from the rhizosphere soil samples with the E.Z.N.A.® soil DNA Kit (Omega Bio-Tek, Norcross, GA, United States), and the quality of the extracted DNA was checked by NanoDrop 2000 UV–Vis spectrophotometer. The bacterial universal V3–V4 region of the 16S rRNA gene was amplified by polymerase chain reaction (PCR) using primers 338F (5′-ACTCCTACGGGAGGCAGCAG-3′) and 806R (5′-GGACTACHVGGGTATCTAAT-3′; see Supplementary Table S1 for the DNA metabarcoding details). The PCR products were quantified using Quantus™ Fluorometer (Promega Corporation, Madison, United States) after purification. The purified amplicons were mixed in equimolar amounts, and sequenced by Majorbio Bio-Pharm Technology Co. Ltd. (Shanghai, China) on the Illumina MiSeq PE300 platform (Illumina Inc., San Diego, CA, United States). More details on the DNA metabarcoding molecular analyses can be found in Supplementary Table S1. All sequences have been deposited in the NCBI SRA database under accession number SRA data: PRJNA884485.

### 2.5. Data analysis

Calculations were performed using Microsoft Excel, and statistical analyses were performed using the DPS Statistics 18.10 software.^1^ All analyses were conducted on the Majorbio Cloud Platform^2^; for instance, α-diversity was calculated using the Mothur software (v.1.30.2); rarefaction curves were generated using Mothur at a 97% identity level; Venn and bar diagrams were generated with R script (v.3.3.1); and Circos plot was visualized using Circos-0.67-7.^3^ Beta diversities were visualized using principal coordinate analysis (PCoA), based on the distance matrix, with bray_curtis. Redundancy analysis (RDA) was conducted using R (version 3.3.1) rda analysis, and graphed using the *vegan* package. Finally, network analysis was performed using the Networkx software. Data are presented as mean ± standard error followed by Duncan’s multiple range test, and differences among the means of different treatments were determined at *p* < 0.05.

## 3. Results

### 3.1. Biochar- and BOF-induced changes in soil physicochemical and plant properties

The effects of biochar and BOF application on the physical and chemical properties of soil are summarized in Table 1. All the physicochemical properties of soil, except pH, were significantly increased by the biochar and BOF treatments. Except BOF, all treatments significantly reduced the VW and increased the TN of soil compared with the CK. All treatments, except FB and BOF, significantly increased the soil AN content compared with the CK. Compared with the CK, the CB treatment significantly reduced the AP, while the other treatments significantly increased the AP content of soil. The AK content of all plots receiving BOF (FB1_BOF, FB2_BOF, CB1_BOF, and CB2_BOF) was significantly higher than that of plots not treated with BOF (FB, CB, and CK). Among all treatments, CB2_BOF and FB2_BOF showed the highest AK level. Compared with CK, all treatments significantly increased the soil OC and OM contents, and significantly promoted crop growth (*p* < 0.05); FB2_BOF caused the greatest increase in plant height (from 123.00 to 154.00 cm; Supplementary Figure S1).

**Table 1:** Effect of biochar and BOF on the physical and chemical properties of soil.

	CK	CB	FB	BOF	CB1-BOF	CB2-BOF	FB1-BOF	FB2-BOF
VW	1.61 ± 0.04a	1.45 ± 0.05b	1.4 ± 0.03b	1.6 ± 0.01a	1.44 ± 0.01b	1.38 ± 0.03b	1.39 ± 0.01b	1.42 ± 0.02b
pH	8 ± 0.15a	7.94 ± 0.15a	7.96 ± 0.14a	7.97 ± 0.13a	7.96 ± 0.08a	7.97 ± 0.09a	7.97 ± 0.12a	7.96 ± 0.1a
TN	1.37 ± 0.04e	1.7 ± 0.05d	1.72 ± 0.05d	1.32 ± 0.03e	1.95 ± 0.11c	1.83 ± 0.13 cd	2.27 ± 0.1b	3.27 ± 0.12a
AN	71.6 ± 4.25e	83.07 ± 5.16d	71.41 ± 3.4e	70.56 ± 2.91e	77.56 ± 6.33de	103.5 ± 7.31c	116.96 ± 6.75b	133.81 ± 7.63a
AP	4.52 ± 0.17 g	2.87 ± 0.11 h	5.86 ± 0.21f	8.19 ± 0.35e	16.29 ± 1.08d	20.37 ± 0.64b	17.66 ± 0.56c	21.5 ± 1.23a
AK	270.68 ± 16.46d	277.6 ± 15.2d	280.31 ± 14.11d	459.75 ± 19.38c	535.12 ± 25.77b	582.57 ± 26.51a	488.53 ± 21.63c	576.98 ± 24.6a
OC	8.77 ± 0.37 g	13.37 ± 0.73e	12.51 ± 0.83ef	11.11 ± 0.78f	16.03 ± 1.26d	18.31 ± 1.45c	25.58 ± 1.74b	30.14 ± 2.34a
OM	15.46 ± 1.23 g	22.71 ± 0.91e	21.24 ± 1.57ef	18.82 ± 0.91f	26.64 ± 2.89d	31.23 ± 2.21c	43.09 ± 2.88b	50.95 ± 3.15a

Data represent mean ± standard deviation (SD) of three biological replicates. Different lowercase letters within a row indicate significant differences (*P* < 0.05).

**Figure 1:**
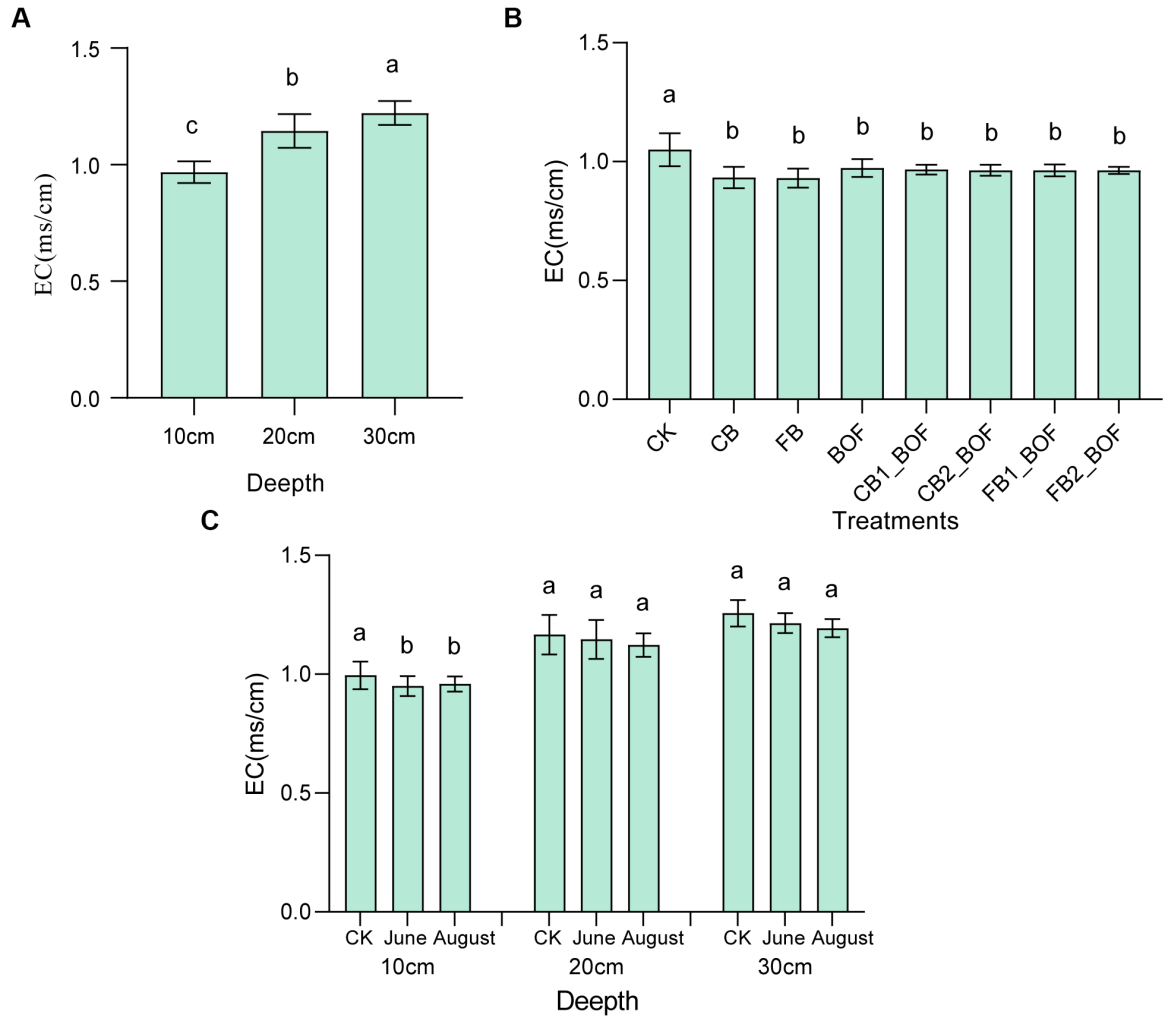
Effect of biochar and BOF on soil EC. **(A)** Comparison of soil conductivity at different depths. **(B)** Comparison of conductivity among different treatments. **(C)** Comparison of conductivity in different periods. CK, soil without amendment; CB, soil amended with 10 mesh biochar; FB, soil amended with 30 mesh biochar; BOF, soil amended with BOF; CB1_BOF (100 t ha^−1^ 10 mesh biochar + BOF), CB2_BOF (150 t ha^−1^ 10 mesh biochar + BOF), FB1_BOF (100 t ha^−1^ 30 mesh biochar + BOF), FB2_BOF (150 t ha^−1^ 30 mesh biochar + BOF); Error bars represent the standard error of mean (*n* = 3). Different lowercase letters indicate significant differences among treatments (*p* < 0.05).

Biochar and BOF induced significant changes in soil EC (Figure 1). All treatments significantly decreased the EC by 7%–11.58%, regardless of whether biochar and BOF were applied alone or in combination, however, no significant difference was observed among treatments, moreover, this reduction was only observed at 10 cm depth, and no significant difference was detected among CK and treatments at 20 or 30 cm depth. Again, compared to before planting, soil EC can be significantly reduced by an average of 4% after planting at 10 cm depth, but not at 20 cm or 30 cm depth. In addition, soil EC at 10 cm depth was significantly lower than that of 20 cm, and 20 cm depth was significantly lower than that of 30 cm depth.

### 3.2. Sequence data and bacterial composition

After filtering out low-quality reads, a total of 1,387,916 high-quality reads were obtained. The total number of bases was 576,080,099, and the average read length was 415.13 bp. Rarefaction curves tended to approach the saturation plateau in all 24 samples (Supplementary Figure S2), combined with the richness and diversity indices (Supplementary Table S2), suggesting that the data were sufficiently large to capture most of the bacterial diversity in the samples. The number of OTUs was highest in the FB treatment and lowest in the CB1_BOF treatment.

**Figure 2:**
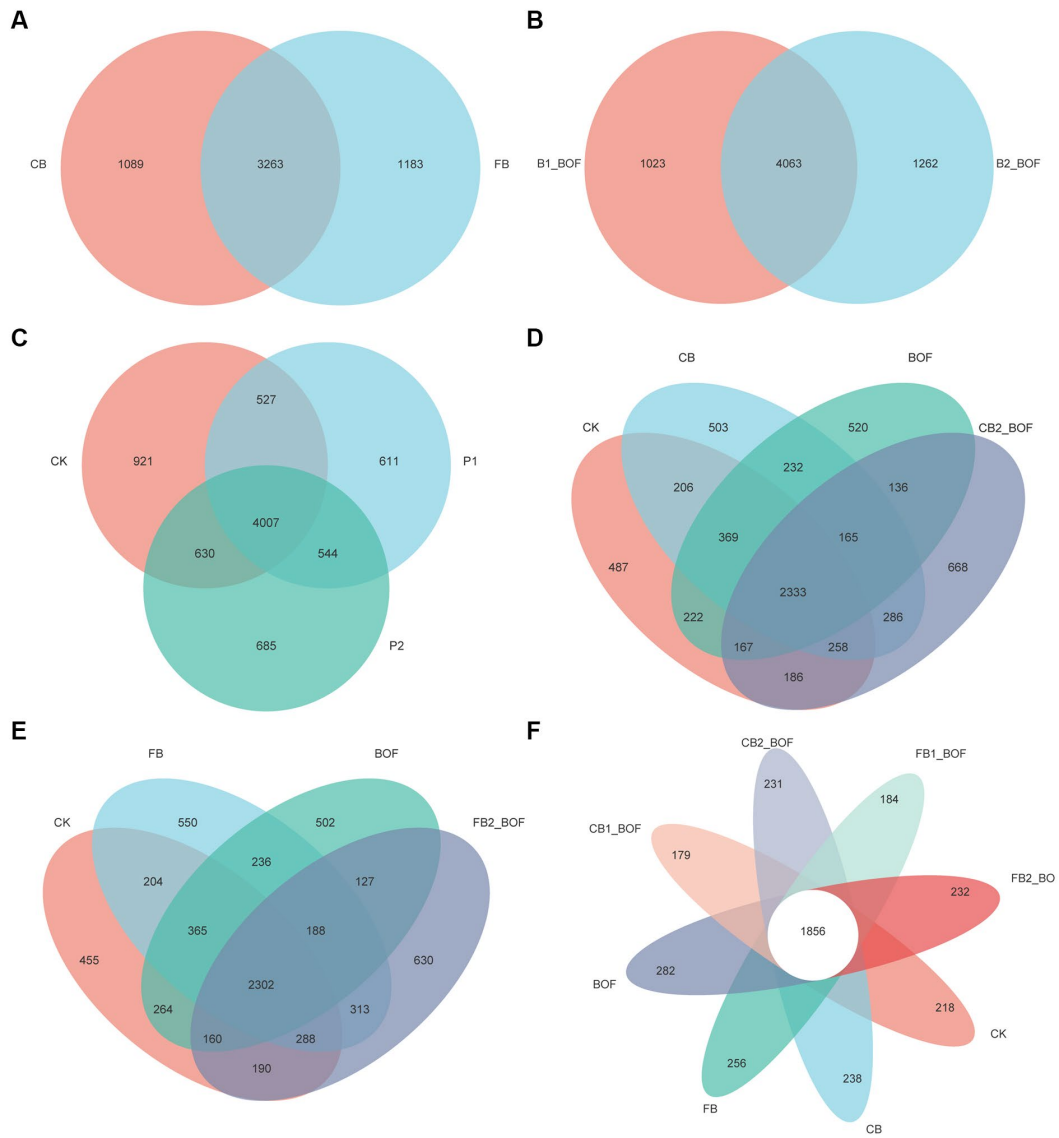
Venn diagrams showing the number of OTUs identified in different treatments. **(A)** Grouping by particle size of biochar. **(B)** Grouping by different biochar rate. **(C)** Grouping by different plant stage. **(D)** Grouping by coarse biochar and BOF. **(E)** Grouping by fine biochar and BOF. **(F)** Grouping by all treatments.

The number of common and unique bacterial OTUs in the different samples is shown in Figure 2. Treatments receiving biochar of different particle sizes shared 3,263 of the total 5,535 OTUs (Figure 2A), and those receiving biochar at different application rates shared 4,063 of the total 6,348 OTUs (Figure 2B). A total of 7,925 OTUs were detected across all libraries, with 4,007 OTUs common to different plant growth periods (Figure 2C), with the higher number of unique OTUs obtained in the CK (921). In addition, in the comparison of two groups of different particle sizes of biochar, the CK groups, biochar only, BOF only, and biochar + BOF treatments showed a similar trend, with the biochar + BOF treatment harboring the highest and CK possessing the lowest number of unique OTUs, regardless of the particle size, and shared 2,333 (CB) and 2,302 (FB) OTUs, respectively (Figures 2D,E). Additionally, all treatments shared 1,856 OTUs, with the BOF treatment containing the highest number of unique OTUs (Figure 2F).

### 3.3. Taxonomic analysis of soil microbiota

**Figure 3:**
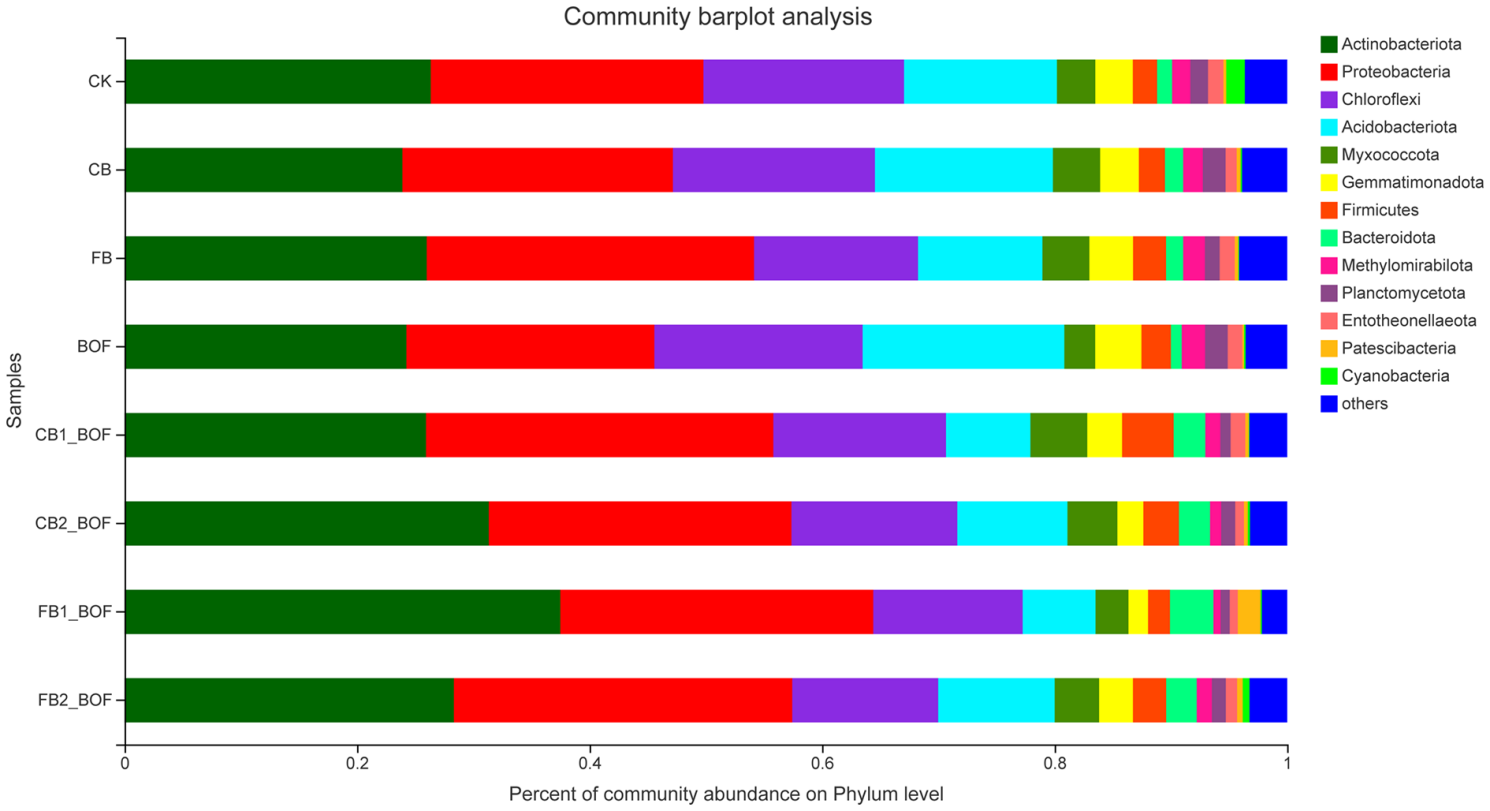
Relative abundance of bacteria at the phylum level in each sample.

The 7,925 OTUs were classified into 45 phyla, 139 classes, 339 orders, 580 families, 1,154 genera, and 2,360 species. High-throughput sequencing revealed the diversity of bacterial communities in different samples at the phylum level (Figure 3). Actinobacteriota and Proteobacteria were the most dominant bacterial phyla, accounting for more than 40% of the bacterial population in each sample, followed by Chloroflexi and Acidobacteriota. Proteobacteria was the predominant phylum in FB, CB1_BOF, and FB2_BOF treatments, while Actinobacteriota was the predominant phylum in all other treatments. Compared with CK, the relative abundance of Proteobacteria and Bacteroidota, two of the top 10 phyla, increased in the biochar + BOF treatment; and Myxococcota, Gemmatimonadota, Firmicutes, Bacteroidota, and Methylomirabilota increased in the biochar only treatment; and Chloroflexi Acidobacteriota, Gemmatimonadota, Firmicutes, Methylomirabilota, and Planctomycetota increased in the BOF only treatment.

Actinobacteria was the predominant class in CB2_BOF and FB1_BOF, while Alphaproteobacteria was the dominant class in the other treatments. Micromonosporales occupied the top of the relative abundance list of the order in CB2_BOF and FB1_BOF; Vicinamibacterales was the predominant order in CB and BOF, and Tistrellales was the most abundant order in the other treatments. Micromonosporaceae was the most abundant family in CB2_BOF and FB1_BOF, while norank_f__Geminicoccaceae was the most abundant family in the other treatments; similarity, *Micromonospora* was the most abundant genus in CB2_BOF and FB1_BOF, and *norank_f_Geminicoccaceae* was the predominant genus in the other treatments (Supplementary Table S3).

**Figure 4:**
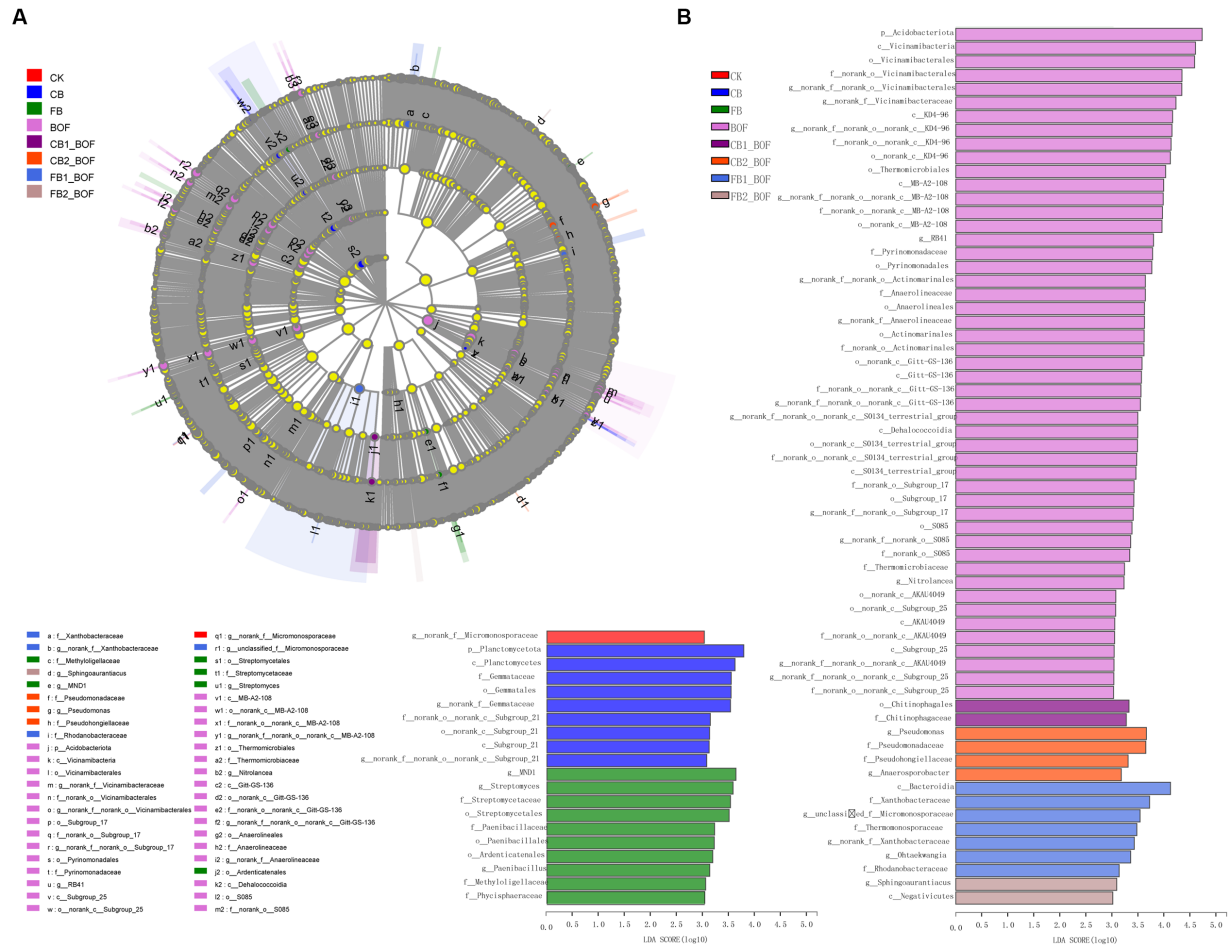
LEfSe and LDA. **(A)** LEfSe. **(B)** LDA.

Significantly different taxa showed high abundance in the different treatments, as determined by Linear discriminant analysis Effect Size (LEfSe; Figure 4). In CK, only *norank_f__Micromonosporaceae* was enriched at the genus level. On the contrary, in BOF, 1 phylum, 8 classes, 13 orders, 13 families, and 13 genera were enriched, namely, Acidobacteriota (from phylum to genus); MB-A2-108, Gitt-GS-136, Dehalococcoidia, KD4-96, and S0134_terrestrial_group (from class to genus); Thermomicrobiales (from order to genus). In addition, in CB, one phylum, two classes, two orders, two families, and two genera were enriched, namely, Planctomycetota (from phylum to genus) and Subgroup_21 (from class to genus). In FB, three orders, four families, and five genera were enriched, including Paenibacillales and Streptomycetales (from order to genus), Ardenticatenales (enriched only at the order level), Methyloligellaceae and Phycisphaeraceae (enriched only at the family level), and *MND1* (enriched only at the genus level). In CB1_BOF, only Chitinophagales (at the order level) and Chitinophagaceae (at the family level) were enriched. In CB2_BOF, two families and two genera were enriched, including Pseudomonadales (from family to genus), Pseudohongiellaceae (at the family level), and *Anaerosporobacter* (at the genus level). In FB1_BOF, one class, three families, and three genera were enriched, including Xanthobacteraceae (from family to genus), Bacteroidia (at the class level), Thermomonosporaceae and Rhodanobacteraceae (at the family level), and *unclassified_f__Micromonosporaceae* and *Ohtaekwangia* (at the genus level). In FB2_BOF, only Negativicutes (at the class level) and Sphingoaurantiacus (at the genus level) were enriched. These differentially abundant taxa could be considered as potential biomarkers (LDA > 3, *p* < 0.05).

### 3.4. β-Diversity analysis

**Figure 5:**
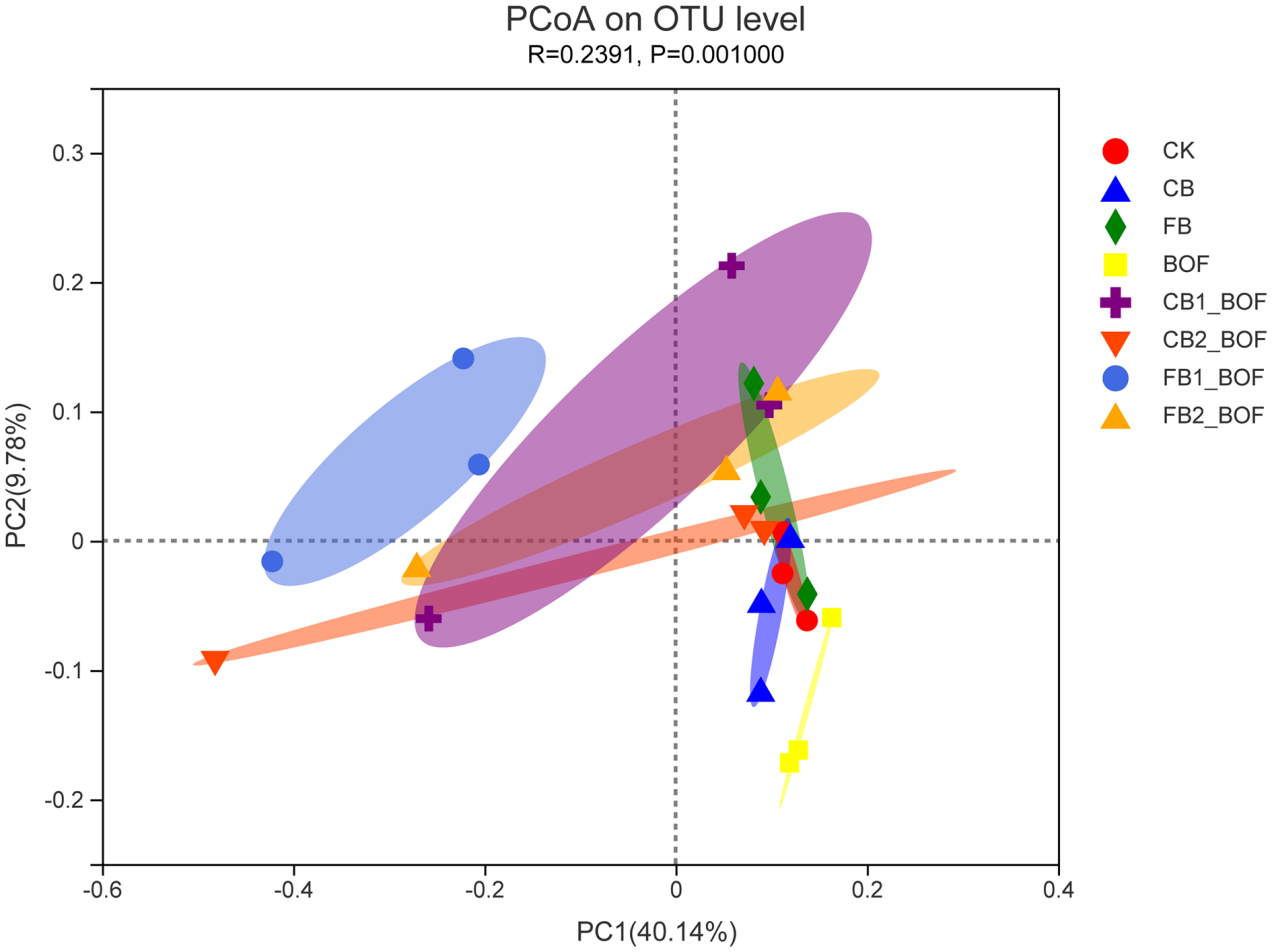
PCoA of the relationship among treatments, based on the similarity in bacterial community composition The first two components (PCoA1 and PCoA2) account for 49.92% of the variation in bacterial community composition.

PCoA was conducted to further identify the microbial populations associated with biochar and BOF (Figure 5). This analysis revealed the main variations in bacterial community composition and abundance among the treatments. The biochar + BOF treatments showed a lower PC1 value (40.14%), while the BOF treatments showed a lower PC2 value (9.78%; *p* = 0.001). In addition, the treatments with different biochar contents and particle sizes clustered together separately, indicating that both biochar content and particle size affected the microbial community structure, while the biochar only treatment was closer to CK. PCoA analysis revealed that the BOF treatment had the greatest effect on bacterial community structure, followed by biochar content and particle size.

### 3.5. Relationship between environmental parameters and microbial communities

**Figure 6:**
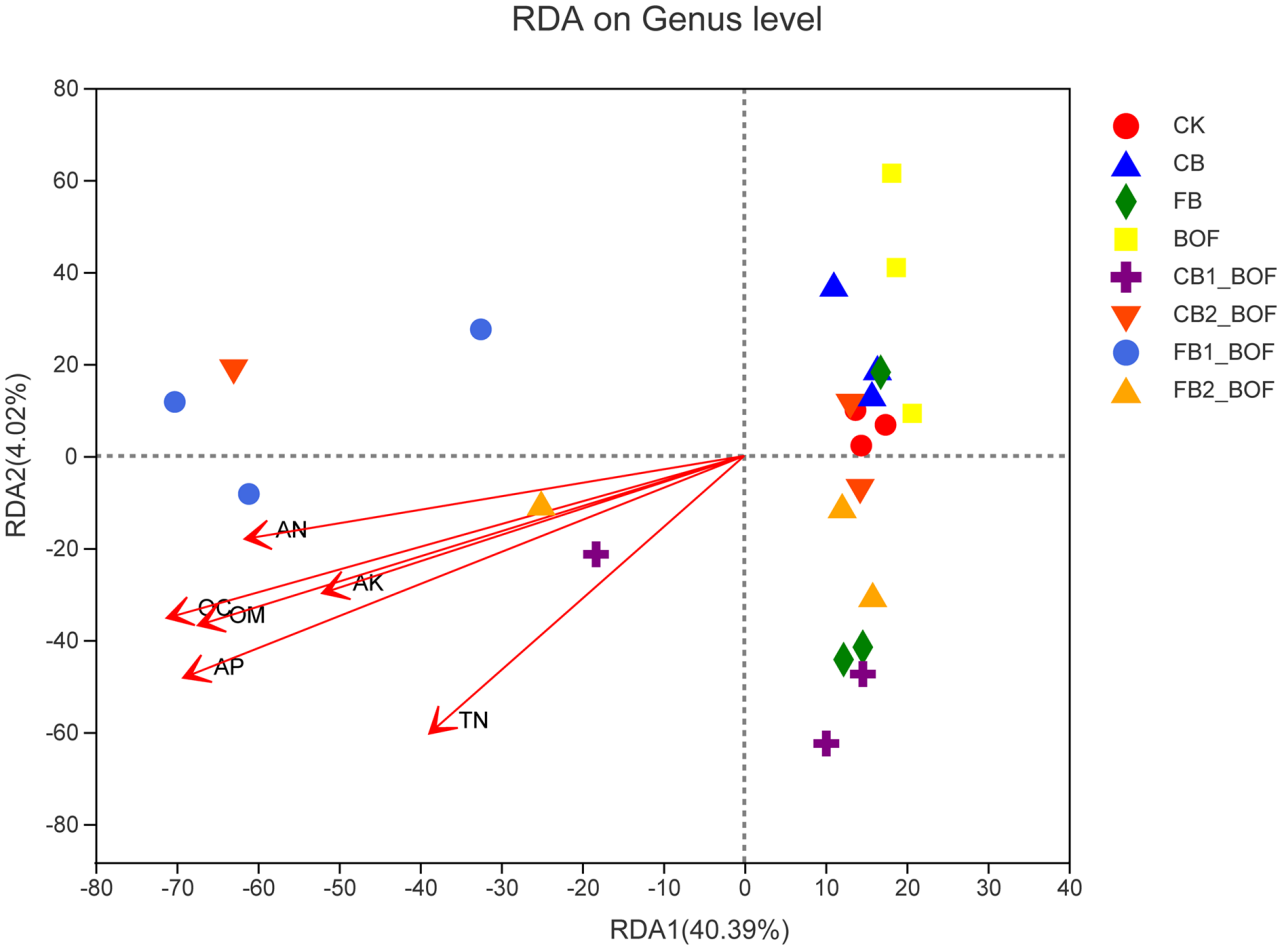
RDA of the relationship between soil characteristics and soil bacterial community. The first two components (PCoA1 and PCoA2) account for 44.41% of the variation in bacterial community composition.

The RDA results revealed the relationship of bacterial community composition with AP, AN, TN, AK, OC, and OM (Figure 6). The first axis accounted for 40.39% of the overall variation in microbial community composition, while the second axis accounted for only 4.02%. RDA showed that the bacterial community structure in biochar + BOF treatments was positively correlated with AP, AK, OC, and OM.

### 3.6. Network structure

**Figure 7:**
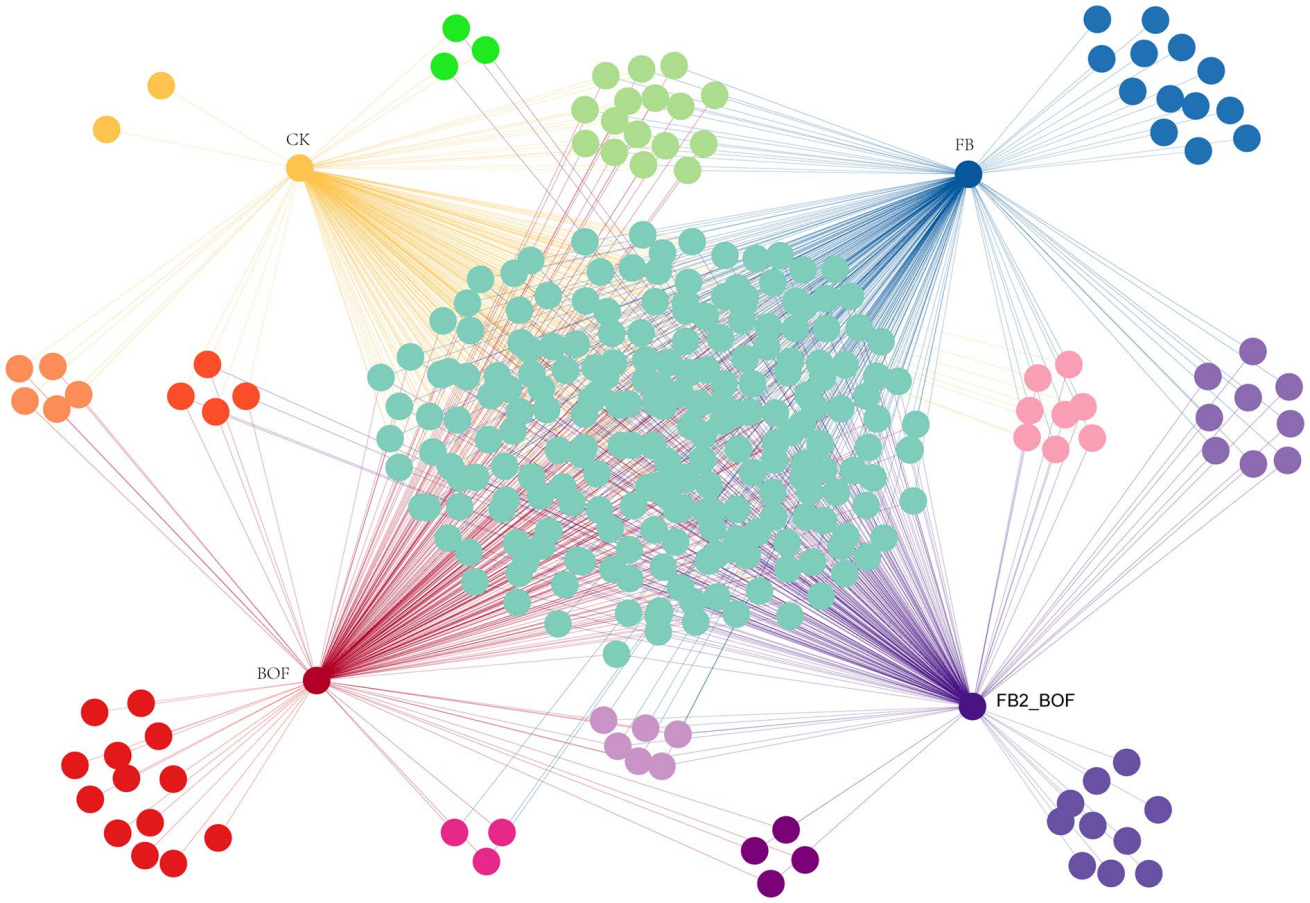
Co-occurrence network analysis of microbial communities.

A bipartite association network was used to visualize the associations among treatments at the order level (Figure 7). Compared with the CK, which contained only unique orders, the FB, BOF, and FB2_BOF treatments harbored a greater number of unique orders, on the basis of 220 shared orders. FB and FB2_BOF shared nine unique orders, which was the highest number of orders among two-way interactions, however, no order was shared between CK and FB. In addition, CK, FB, and BOF shared 13 orders, which was the highest number of orders among three-way interactions.

**Figure 8:**
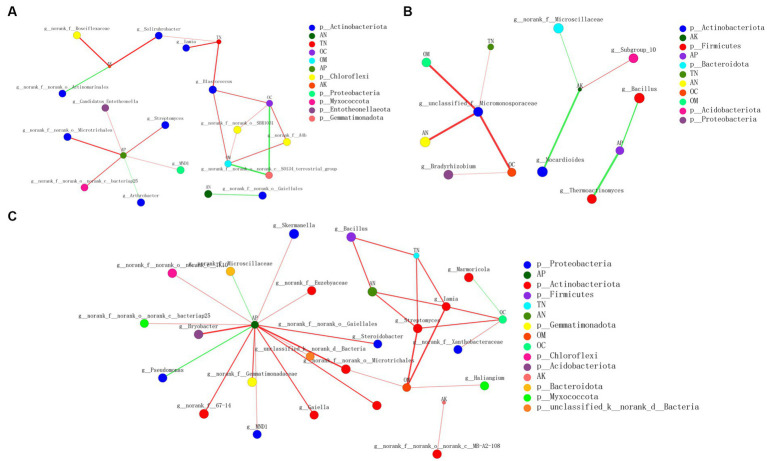
Correlation network analysis of environmental parameters and microbial communities **(A)** CB-FB. **(B)** CB1_BOF-FB1_BO F. **(C)** CB2_BOF-FB2_BOF.

Correlation network analysis was conducted to explore the complexity of the interactions among the communities in different treatments and environmental parameters and to assess their topological properties (Figure 8). The complexity of CB2_BOF-FB2_BOF was greater than those of CB-FB and CB1_BOF-FB1_BOF, indicating that both the addition of BOF and higher application rate of biochar increased the complexity of the correlation between microorganisms and soil environmental factors. The average number of connections per node was higher following the CB2_BOF-FB2_BOF treatment (node average degree = 2.1) than after the CB-FB (node average degree = 2) or CB1_BOF-FB1_BOF (node average degree = 1.5) treatment (Supplementary Table S4). CB2_BOF-FB2_BOF treatment also resulted in a higher number of positive correlations (positive edges = 27) than CB-FB (positive edges = 16) or CB1_BOF-FB1_BOF (positive edges = 6). Nodes with the highest connections between environmental parameters and bacteria were AP (6) and *Blastococcus* (3) in CB-FB, respectively; AK (3) and *unclassified_f__Micromonosporaceae* (4) in CB1_BOF-FB1_BOF, respectively; AP (15) and *Iamia* (4) in CB2_BOF-FB2_BOF, respectively.

## 4. Discussion

Biochar (Xie et al., 2015), inorganic fertilizer (Chen et al., 2021), organic fertilizer (Moraditochaee et al., 2014; Gao et al., 2020), microbial fertilizer (García-Fraile et al., 2015; Milton et al., 2020), and BOF (Jena et al., 2020) improve soil physicochemical properties and enhance plant growth. Especially microbial fertilizer, which is considered to be one of the most promising means to improve the productivity of the crop in an environment-friendly manner. Microbes present in microbial fertilizers play a pivotal role in optimizing various activities like organic matter decomposition, plant nutrient absorption such as that of potassium, nitrogen, phosphorous in plants (Milton et al., 2020; Chauhan S. et al., 2022; Rani et al., 2022; Upadhyay et al., 2022b). Several studies have shown that the co-application of biochar and other fertilizers is better than the application of biochar or fertilizer alone (Naeem et al., 2017; Li et al., 2018; Cui et al., 2020; Bai et al., 2022). However, fewer studies performed field experiments to investigate the reclamation of saline-alkaline land through the co-application of biochar and BOFs. Our results demonstrate that the combined application of biochar and BOF is more effective than the application of either biochar or BOF alone for enhancing plant growth and soil physicochemical properties, and the BOF only treatment is superior to the biochar only treatment. Several factors may be responsible for these results. First, the enhancement of soil properties is partly dependent on the nutrient supply from biochar and BOF (Agegnehu et al., 2017). Second, biochar has a porous structure and is therefore able to store and control the subsequent slow-release of nutrients, which improves soil fertility, aggregation, and water storage capacity, increases nutrient contents, and decreases nutrient leaching (El-Naggar et al., 2019). Third, the functions of BOF and biochar were amplified when applied together, probably because BOF increased the diversity of beneficial microorganisms, while biochar provided a better living environment for microorganisms owing to its porous nature (Cui et al., 2021). Therefore, compared with the application of either biochar or BOF, the co-application of biochar and BOF promotes microbial activity and improves soil physicochemical properties, in turn enhancing plant growth.

The effect of biochar application rates on crop yield varies considerably, with some application rates even having a negative effect (Biederman and Harpole, 2013; Chen et al., 2018). In general, increasing biochar application rate increases crop yield; for example, an application rate of >30 t ha^−1^ was better than that of <30 t ha^−1^ (Bai et al., 2022). Yao et al. (2017) reported an increase in the soil OM and OC contents after the application of maize stalk-derived biochar at rates of 50, 100, and 200 Mg ha^−1^. Cui et al. and Zheng et al. also proved that 3%–5% biochar application enhanced the *S. cannabina* height and biomass (Zheng et al., 2018; Cui et al., 2021). Our results were consistent with the above reports. Higher application rates lead to increased costs, but the cost can be reduced by applying the amendment around the root zone of the plant. Soil salinity, and the associated soil compaction and low fertility, is a major problem for land management in the coastal zone (Xiao and Meng, 2020). In the current study, the soil was silty clay and was heavily compacted, leading to low aeration and water permeability of the surface soil, which directly inhibits the growth of plant roots and the absorption of nutrients. Therefore, the addition of porous biochar greatly improved the soil structure, increased soil permeability, and reduced soil VW, which was consistent with Xiao and Meng (2020) and Gu et al. (2022). Moreover, the improved soil porosity by biochar addition due to the abundant pores in the biochar, beneficial for air and water infiltration could be one of the reasons responsible for the enhanced plant growth (Zheng et al., 2018). In the current study, the application of biochar alone or together with BOF significantly reduced the soil VW, while the application of BOF alone did not have this effect. This is undoubtedly due to the porous structure of biochar, which makes biochar an excellent conditioner for compacted soil. Additionally, the biochar particle size and application rate in this study had no significant effect on soil VW among different treatments, this may be due to the fact that the rate and particle size of biochar used in this study did not reach a point where there was a significant difference.

Although the particle size of biochar is an important factor affecting soil characteristics and functions (Anders et al., 2013; Xie et al., 2015; Liao and Thomas, 2019), few studies have been conducted on this topic. In the current study, finer particle size and higher application rate of biochar showed the best results when applied together with BOF, but application of finer particle-size biochar alone only improved soil AP content, confirming that biochar with fine particle significantly increased the release of P (Sarfraz et al., 2020). The particle size of biochar affects soil water storage by changing the pore space between particles (interpores) and by adding pores that are part of the biochar (intrapores; Liu et al., 2017). Thus, finer particle-size biochar has larger interpores and intrapores, providing more area for nutrients and microbial populations, thus amplifying the function of BOF. In addition, fine particles of biochar can be more readily degraded by microbes than coarse particles (Chen et al., 2017). Therefore, finer biochar together with BOF improve the physical and chemical properties of soil and promote plant growth. Nonetheless, different plant species respond slightly differently to biochar particle size (Kartika et al., 2018). Therefore, intensive studies are needed to explore the appropriate biochar particle size for each plant species in saline-alkaline soil for a more comprehensive understanding.

Biochar has a strong influence on soil salinity (Saifullah Dahlawi et al., 2018). For instance, in a pot experiment conducted for 3 years using rice plants, the application of biochar decreased the value of soil EC by 28.96% (Huang et al., 2022). Yue et al. (2016) demonstrated that biochar can also improve the leaching of soluble salts to decrease soil EC. However, biochar application at high rates has been shown to increase salinity and/or sodicity (Zheng et al., 2018). In the current study performed using *S. cannabina*, the application of biochar with or without BOF significantly reduced the EC of saline-alkaline soil at 10-cm depth, regardless of its particle size or application rate; however, no effect on soil EC was observed at 20-cm or greater depth. Biochar alleviates salt stress by modifying soil properties and regulating soil bacterial abundance and community structure (Huang et al., 2022). Similarly, salt-tolerant plant cultivation and fertilizer application have also been claimed to decrease salt stress and improve plant yield in saline soils (Qadir et al., 2007). *Sesbania cannabina* is well-adapted to salinity stress (Ren et al., 2016), and therefore can significantly reduce EC after plantation. The same effect can be achieved by supplementation with biochar and BOF, based on the above reasons, although no significant differences were detected among treatments. The reason why a significant reduction in EC was observed at only a depth of 10 cm below the surface, and not at greater depths, was probably because the capillary roots and nodules of *S. cannabina* roots were mainly distributed in the soil above 10 cm. This is consistent with the study of Ma et al. (2012), who reported that the maximum reduction in soluble salt and sodium in saline soil occurs at a depth of 15–20 cm below the surface, where the fibrous root system of hybrid giant Napier grass was the most dense. This can be explained based on the fibrous root system of plants, which forms micro/macro pores in the soil, improving the hydraulic conductivity of the soil and increasing water availability for plant growth. In addition, the distribution of fibrous roots in the soil promotes the adsorption, transport, and biometabolism of ionic substances, leading to nutrient accumulation and soluble salt uptake.

Accumulating evidence suggests that biochar and BOF affect soil microorganisms, which play vital roles in the utilization of soil nutrients, suppression of pests and diseases, and promotion of plant growth (Qiu et al., 2019; Jena et al., 2020; Alkharabsheh et al., 2021). Compared with the CK, the unique OTUs identified after planting *S. chinensis* were less (Figure 2C), and all biochar treatments, regardless of the particle size, application rate, and the presence or absence of BOF, harbored more unique OTUs (Figures 2D,E), indicating that biochar and BOF induce great changes in soil microbiota. Actinobacteriota, Proteobacteria, and Chloroflexi accounted for 63–77% of all sequences in the soil samples. However, the relative abundance of these phyla varied among the different treatments, which was partly consistent with Huang et al. (2022). Awasthi et al. (2017) reported that Proteobacteria play an important role in OM degradation, which is related to the carbon cycle. Actinobacteria are reportedly associated with the degradation of recalcitrant polymers, and are thus considered important for the turnover of soil OM (Zhou et al., 2018). Acidobacteria and Chloroflexi are oligotrophic bacteria that can survive in low-nutrient soil, whereas Proteobacteria prefer nutrient-rich soil (Huang et al., 2022). This may explain why the relative abundance of Proteobacteria in four biochar + BOF treatments conducted in this study was higher than their abundance in the CK. However, the relative abundance of Chloroflexi was lower in the other treatments than in the CK. This is consistent with the results of RDA, which showed that relative bacterial abundance was positively correlated with AK, AP, OC, and OM following the co-application of biochar and BOF; this is partly consistent with previous studies (Gao et al., 2020; Ren et al., 2020).

*Bacillus* species are commercially marketed as biopesticides, biofertilizers, and soil amendments (Cao et al., 2011). It is worth noting that the abundance of *Bacillus* species in the rhizosphere soil did not increase after the application of *Bacillus* compound biofertilizer (Gu et al., 2022; Zhu et. al., 2020). In the current study, *Bacillus* also did not flourish with the application of BOF, as expected. This may be owing to the competitive relationship between *Bacillus* and other bacteria in saline-alkali soil. In this study, *Bacillus* was positively correlated with TN and AN, and negatively correlated with AP. *Iamia*, which belongs to the Actinobacteriota phylum, produces antibiotics to help plants resist pathogen infection (Jin et al., 2016; Liu et al., 2016; Zhang H. Q. et al., 2022). Wang et al. (2021) reported that SOM, AP, and AK were positively correlated with *Iamia*. In the current study, the relative abundance of *Iamia* was positively correlated with OC, OM, TN, and AN, which is partly consistent with the results of Wang et al. (2021). The relative abundance of *Iamia* was significantly higher in 0.6% and 1.2% biochar treatments than in the no biochar treatment (He et al., 2021). Additionally, the relative abundance of *Iamia* was higher in biochar treatments than in the CK. In addition, *Iamia* was demonstrated that indirectly positively correlated with OM, TN, and AN (Chen et al., 2012; Xu et al., 2018). *Xanthobacteraceae* is closely involved in P solubilization and N fixation (Zhang et al., 2021), exhibits an associative relationship with microbial N (Obermeier et al., 2020), and can fix N (Lee et al., 2005; Yang et al., 2021). However, in the current study, *Xanthobacteraceae* was positively correlated with OC, and was enriched in FB1_BOF. This may be consistent with the results of Xu et al. (2022), who reported that *Xanthobacteraceae* significantly increased in response to organic mulch. *Streptomyces*, an important group of soil bacteria belonging to the actinomycetes family, are widely reported in the literature for their plant growth-promoting rhizobacteria potential (Nassar et al., 2003; El-Tarabily, 2008). Studies have shown that *Streptomyces* can stimulate host plant growth by directly altering hormone balance in the plant, for example, through the production of phytohormones (auxins, cytokinins, and gibberellins), increasing mineral nutrient solubilization (for example, siderophores scavenge ferric iron from the environment), fixing nitrogen, producing cell wall degrading enzymes, and suppressing stress in the plant by producing 1-aminocyclopropane-1-carboxylate (ACC; Wu et al., 2021). In addition, *Streptomyces* can also promote plant growth by causing antagonism toward plant pathogens (Gopalakrishnan et al., 2013). In the present investigation, *Streptomyces was* positively correlated with OC, OM, TN, AN, and AP, and was enriched in FB. Similarly, MND1 which are nitrifying, N-fixing, and cellulose-decomposing bacteria (Zhang D. J. et al., 2022), were also enriched in the FB treatment. Cáceres et al. (2021) also confirmed that genus MND1 (Proteobacteria) was found in soils that are associated with N cycling. In addition, according to Sun et al. (2020), MND1 is significantly positively correlated with AP and TP contents; consistently, our results showed that MND1 was positively correlated with AP. Biochar and BOF changed soil microbial community composition, structure, and abundance, altered microbial habitats, directly or indirectly affected microbial metabolic activities, and provided a more suitable environment for plant-friendly bacteria in the soil, which in turn promoted plant growth. Thus, biochar and BOF could be used as potential soil amendments for improving soil health by altering microbial activities and functions; however, many aspects of biochar and BOF are still to be studied.

## 5. Conclusion

This study provides empirical evidence showing that the application of biochar and BOF is an effective way for improving the rhizosphere soil microecology and crop productivity in saline–alkali soil. Application of biochar and BOF in saline-alkali soil positively influenced the growth of *S. cannabina*. Among all treatments, the impact of 150 t ha^−1^ biochar (30 mesh) application together with BOF was the greatest. Biochar and B_BOF also improved the physical and chemical properties and the nutrient contents of saline-alkaline soil, and altered the diversity and community structure of rhizosphere bacteria in the saline-alkaline soil. The co-application of biochar and BOF along with the planting of *S. cannabina* decreased the EC of soil at a depth of 10 cm below the surface. Overall, biochar and BOF co-application was the most superior, followed by BOF alone and biochar alone. Compared with the lower application rate, the higher application rate was better. Additionally, fine biochar had a stronger effect than coarse biochar. Collectively, our results reinforce the influence of biochar and BOF application on saline-alkaline land in terms of microbial community structure and soil nutrients, providing a suitable method for saline-alkaline land reclamation. Additional studies are needed to investigate the long-term effects and interaction mechanism of biochar, BOF, beneficial microorganisms, and plants.

## Data availability statement

The datasets presented in this study can be found in online repositories. The names of the repository/repositories and accession number(s) can be found in the article/Supplementary material.

## Author contributions

Y-yG contributed to the conception of the study and wrote the manuscript. C-jC and ML contributed to the conception of the study. X-yL, H-yZ, and RF performed the experiments and data analyses. All authors contributed to the article and approved the submitted version.

## Funding

This work was supported by the Sericultural Industry Technical System of Shandong Province (grant no. SDAIT-18-09) the Natural Science Foundation of Shangdong Province, China (ZR2020QC062).

## Conflict of interest

The authors declare that the research was conducted in the absence of any commercial or financial relationships that could be construed as a potential conflict of interest.

## Publisher’s note

All claims expressed in this article are solely those of the authors and do not necessarily represent those of their affiliated organizations, or those of the publisher, the editors and the reviewers. Any product that may be evaluated in this article, or claim that may be made by its manufacturer, is not guaranteed or endorsed by the publisher.

## Footnotes

^1^http://www.dpsw.cn

^2^www.majorbio.com

^3^http://circos.ca/

## References

[ref1] Abukari A. Duwiejuah A. B. (2019). A review of biochar influences on crop outputs and soil assets. Agr. For. J. 3, 74–80. doi: 10.5281/zenodo.3561058

[ref2] Agbna G. Ali A. Bashir A. Eltoum F. Hassan M. (2017). Influence of biochar amendment on soil water characteristics and crop growth enhancement under salinity stress. Int. J. Eng. Works. 4, 49–54. doi: 10.5281/zenodo.555942

[ref3] Agegnehu G. Srivastava A. K. Bird M. I. (2017). The role of biochar and biochar-compost in improving soil quality and crop performance: a review. Appl. Soil Ecol. 119, 156–170. doi: 10.1016/j.apsoil.2017.06.008

[ref4] Alkharabsheh H. M. Seleiman M. F. Battaglia M. L. Shami A. Jalal R. S. Alhammad B. A.. (2021). Biochar and its broad impacts in soil quality and fertility, nutrient leaching and crop productivity: a review. Agronomy 11:993. doi: 10.3390/agronomy11050993

[ref5] Anders E. Watzinger A. Rempt F. Kitzler B. Wimmer B. Zehetner F.. (2013). Biochar affects the structure rather than the total biomass of microbial communities in temperate soils. Agric. Food Sci. 22, 404–423. doi: 10.23986/afsci.8095

[ref6] Aslam R. (2011). A critical review on halophytes: salt tolerant plants. J. Med. Plant Res. 5, 7108–7118. doi: 10.5897/JMPRX11.009

[ref9001] Awasthi M. K. Zhang Z. Q. Wang Q. Shen F. Li R. H. Zhao G. H.. (2017). New insight with the effects of biochar amendment on bacterial diversity as indicators of biomarkers support the thermophilic phase during sewage sludge composting. Bioresource Technol., 238, 589–601. doi: 10.1016/j.biortech.2017.04.100 28482285

[ref7] Bai S. H. Omidvar N. Gallart M. Kämper W. Tahmasbian I. Farrar M.. (2022). Combined effects of biochar and fertilizer applications on yield: a review and meta-analysis. Sci. Total Environ. 808:152073. doi: 10.1016/j.scitotenv.2021.152073, PMID: 34863750

[ref8] Bhardwaj D. Ansari M. W. Sahoo R. K. Tuteja N. (2014). Biofertilizers function as key player in sustainable agriculture by improving soil fertility, plant tolerance and crop productivity. Microb. Cell Fact. 13, 1–10. doi: 10.1186/1475-2859-13-66, PMID: 24885352 PMC4022417

[ref9] Biederman L. A. Harpole W. S. (2013). Biochar and its effects on plant productivity and nutrient cycling: a meta-analysis. GCB Bioenergy 5, 202–214. doi: 10.1111/gcbb.12037

[ref10] Cáceres P. F. F. Vélez L. P. Junca H. Moreno-Herrera C. X. (2021). *Theobroma cacao* L. agricultural soils with natural low and high cadmium (cd) in Santander (Colombia), contain a persistent shared bacterial composition shaped by multiple soil variables and bacterial isolates highly resistant to cd concentrations. Curr Res Microbial Sci. 2:100086. doi: 10.1016/j.crmicr.2021.100086, PMID: 34927107 PMC8649583

[ref11] Cao Y. Zhang Z. Ling N. Yuan Y. Zheng X. Shen B.. (2011). *Bacillus subtilis* SQR 9 can control Fusarium wilt in cucumber by colonizing plant roots. Biol. Fertil. Soils 47, 495–506. doi: 10.1007/s00374-011-0556-2

[ref12] Chauhan S. Mahawar S. Jain D. Udpadhay S. Mohanty S. R. Singh A.. (2022). Boosting sustainable agriculture by Arbuscular Mycorrhiza under stress condition: mechanism and future prospective. Biomed. Res. Int. 2022, 1–28. doi: 10.1155/2022/5275449 PMC9815931 36619307

[ref13] Chauhan P. K. Upadhyay S. K. (2023). Exo-polysaccharide producing bacteria can induce maize plant growth and soil health under saline conditions. Biotechnol. Genet. Eng. Rev., 1–20. doi: 10.1080/02648725.2022.2163812, PMID: 36597411

[ref14] Chauhan P. K. Upadhyay S. K. Tripathi M. Singh R. Krishna D. Singh S. K.. (2022). Understanding the salinity stress on plant and developing sustainable management strategies mediated salt-tolerant plant growth-promoting rhizobacteria and crispr/cas9. Biotechnol Genet Eng., 1–37. doi: 10.1080/02648725.2022.2131958, PMID: 36254096

[ref15] Chen Y. M. Jiao X. G. Wang G. H. Lin Z. H. Sui Y. Y. Fan J.. (2012). Relationship between catalase activities and soil nutrients for farmland phaeozem based on soil removal method. J Engineer Heilongiang Univ. 11, 3–4. doi: 10.13524/j.2095-008x.2012.04.015

[ref16] Chen J. H. Li S. H. Liang C. F. Xu Q. F. Li Y. C. Qin H.. (2017). Response of microbial community structure and function to short-term biochar amendment in an intensively managed bamboo (*Phyllostachys praecox*) plantation soil: effect of particle size and addition rate. Sci. Total Environ. 574, 24–33. doi: 10.1016/j.scitotenv.2016.08.190, PMID: 27621090

[ref17] Chen W. Wang F. Zeng L. Li Q. (2021). Bioremediation of petroleum-contaminated soil by semi-aerobic aged refuse biofilter: optimization and mechanism. J. Clean. Prod. 294:125354. doi: 10.1016/j.jclepro.2020.125354

[ref18] Chen S. F. Zhou Y. Q. Chen Y. R. Gu J. (2018). Fastp: an ultra-fast all-in-one FASTQ preprocessor. Bioinformatics 34, i884–i890. doi: 10.1093/bioinformatics/bty560, PMID: 30423086 PMC6129281

[ref19] Cui Q. Xia J. B. Liu J. T. Yang H. J. Peng L. (2020). Effects of biochar and EM application on growth and photosynthetic characteristics of *Sesbania cannabina* in saline-alkali soil of the Yellow River Delta, China. Chin. J. Appl. Ecol. 31, 3101–3110. doi: 10.13287/j.1001-9332.202009.006, PMID: 33345512

[ref20] Cui Q. Xia J. Yang H. Liu J. Shao P. (2021). Biochar and effective microorganisms promote *Sesbania cannabina* growth and soil quality in the coastal saline-alkali soil of the yellow river delta, China. Sci. Total Environ. 756:143801. doi: 10.1016/j.scitotenv.2020.143801, PMID: 33307496

[ref21] Drake J. A. Cavagnaro T. R. Cunningham S. C. Jackson W. R. Patti A. F. (2016). Does biochar improve establishment of tree seedlings in saline sodic soils? Land Degrad. Dev. 27, 52–59. doi: 10.1002/ldr.2374

[ref22] Duan M. L. Liu G. H. Zhou B. B. Chen X. P. Wang Q. J. Zhu H. Y.. (2021). Effects of modified biochar on water and salt distribution and water-stable macro-aggregates in saline-alkaline soil. J. Soil. Sediment. 21, 2192–2202. doi: 10.1007/s11368-021-02913-2

[ref23] El-Naggar A. Lee S. S. Rinklebe J. Farooq M. Song H. Sarmah A. K.. (2019). Biochar application to low fertility soils: a review of current status, and future prospects. Geoderma 337, 536–554. doi: 10.1016/j.geoderma.2018.09.034

[ref24] El-Tarabily K. A. (2008). Promotion of tomato (*Lycopersicon esculentum* mill.) plant growth by rhizosphere competent 1-aminocyclopropane-1-carboxylic acid deaminase-producing streptomycete actinomycetes. Plant and Soil 308, 161–174. doi: 10.1007/s11104-008-9616-2

[ref25] Fei Y. H. She D. L. Gao L. Xin P. (2019). Micro-CT assessment on the soil structure and hydraulic characteristics of saline/sodic soils subjected to short-term amendment. Soil Tillage Res. 193, 59–70. doi: 10.1016/j.still.2019.05.024

[ref26] Gao C. El-Sawah A. M. Ali D. F. I. Alhaj Hamoud Y. Shaghaleh H. Sheteiwy M. S. (2020). The integration of bio and organic fertilizers improve plant growth, grain yield, quality and metabolism of hybrid maize (*Zea mays* L.). Agronomy 10:319. doi: 10.3390/agronomy10030319

[ref27] García-Fraile P. Menéndez E. Rivas R. (2015). Role of bacterial biofertilizers in agriculture and forestry. AIMS Bioeng. 2, 183–205. doi: 10.3934/bioeng.2015.3.183

[ref9002] Gopalakrishnan S. Srinivas V. Vidya M. S. Rathore A. (2013). Plant growth-promoting activities of streptomyces spp. in sorghum and rice. SpringerPlus, 2, 1–8. doi: 10.1186/2193-1801-2-574 24255867 PMC3825066

[ref28] Gu Y. Y. Zhang H. Y. Liang X. Y. Fu R. Li M. Chen C. J. (2022). Effect of different biochar particle sizes together with bio-organic fertilizer on rhizosphere soil micro-ecological environment on saline-alkali land. Front. Environ. Sci. 10:949190. doi: 10.3389/fenvs.2022.949190

[ref29] Hafez M. Abo El-Ezz S. F. Popov A. I. Rashad M. M. (2021a). Organic amendments combined with plant growth-promoting rhizobacteria (*azospirillum brasilense*) as an eco-friendly by-product to remediate and enhance the fertility of saline sodic-soils in Egypt. Commun. Soil Sci. Plan. 52, 1416–1433. doi: 10.1080/00103624.2021.1885687

[ref30] Hafez M. Popov A. I. Rashad M. M. (2021b). Integrated use of bio-organic fertilizers for enhancing soil fertility–plant nutrition, germination status and initial growth of corn (*Zea Mays* L.). Environ. Technol. Innov. 21:101329. doi: 10.1016/j.eti.2020.101329

[ref31] He X. Xie H. Gao D. Khashi U. Rahman M. Zhou X.. (2021). Biochar and intercropping with potato–onion enhanced the growth and yield advantages of tomato by regulating the soil properties, nutrient uptake, and soil microbial community. Front. Microbiol. 12:695447. doi: 10.3389/fmicb.2021.695447, PMID: 34512573 PMC8429823

[ref32] Hidri R. Barea J. M. Mahmoud M. B. Abdelly C. Azcón R. (2016). Impact of microbial inoculation on biomass accumulation by *Sulla carnosa* provenances, and in regulating nutrition, physiological and antioxidant activities of this species under non-saline and saline conditions. J. Plant Physiol. 201, 28–41. doi: 10.1016/j.jplph.2016.06.013, PMID: 27393918

[ref33] Huang J. Zhu C. Kong Y. Cao X. Zhu L. Zhang Y. C.. (2022). Biochar application alleviated rice salt stress via modifying soil properties and regulating soil bacterial abundance and community structure. Agronomy 12:409. doi: 10.3390/agronomy12020409

[ref34] Hunt J. DuPonte M. Sato D. Kawabata A. (2010). The basics of biochar: a natural soil amendment. Soil Crop Man. 30, 1–6.

[ref35] International Biochar Initiative (IBI). (2015). Standardized product definition and product testing guidelines for biochar that is used in soil. Version 1.1. Available at https://www.biochar-international.org/wp-content/uploads/2018/04/IBI_Biochar_Standards_V2.1_Final.pdf

[ref36] Jena P. Bisarya D. Kumar V. (2020). Role of bio fertilizer in crop production (an element of sustainable agriculture): a review. Int. J. Chem. Stud. 8, 44–49. doi: 10.22271/chemi.2020.v8.i5a.11009

[ref37] Jia L. Wei R. Jiang H. Yang X. Shen Q. (2010). Application of bio-organic fertilizer significantly affected fungal diversity of soils. Soil Sci. Soc. Am. J. 74, 2039–2048. doi: 10.2136/sssaj2009.0437

[ref38] Jin D. Kong X. Li H. Luo L. Zhuang X. Zhuang G.. (2016). *Patulibacter brassicae* sp. nov., isolated from rhizosphere soil of Chinese cabbage (*Brassica campestris*). Int. J. Syst. Evol. Microbiol. 66, 5056–5060. doi: 10.1099/ijsem.0.001469, PMID: 27620694

[ref39] Kartika K. Lakitan B. Wijaya A. Kadir S. Widuri L. I. Siaga E.. (2018). Effects of particle size and application rate of rice-husk biochar on chemical properties of tropical wetland soil, rice growth and yield. Aust. J. Crop. Sci. 12, 817–826. doi: 10.21475/ajcs.18.12.05.PNE1043

[ref40] Lee K. B. Liu C. T. Anzai Y. Kim H. Aono T. Oyaizu H. (2005). The hierarchical system of the ‘*Alphaproteobacteria*’: description of *Hyphomonadaceae* fam. Nov., *Xanthobacteraceae* fam. Nov. and *Erythrobacteraceae* fam. Nov. Int. J. Syst. Evol. Microbiol. 55, 1907–1919. doi: 10.1099/ijs.0.63663-0, PMID: 16166687

[ref41] Li Z. R. Li D. H. Zhou S. F. Zhou X. M. Bai L. Y. (2018). The weeds control of a novel bioorganic fertilizer and its effects on agronomic traits of rice. Int. J. Agric. Biol. 20, 507–512. doi: 10.17957/IJAB/15.0509

[ref42] Li Q. Q. Xu C. Y. Geng Z. C. Zhang J. C. Chen S. L. Dong S. H. (2019). Impact of biochar on soil bulk density and aggregates of lou soil. Environ. Sci. 40, 3388–3396. doi: 10.13227/j.hjkx.201808094 31854742

[ref43] Li Y. Yan J. Yu B. Wang E. T. Li X. Yan H.. (2016). *Ensifer alkalisoli* sp nov isolated from root nodules of *Sesbania cannabina* grown in saline-alkaline soils. Int. J. Syst. Evol. Microbiol. 66, 5294–5300. doi: 10.1099/ijsem.0.001510, PMID: 27653171

[ref44] Liao W. Thomas S. C. (2019). Biochar particle size and post-pyrolysis mechanical processing affect soil pH, water retention capacity, and plant performance. Soil Syst. 3:14. doi: 10.3390/soilsystems3010014

[ref45] Liu H. Kumar V. Yadav V. Guo S. S. Sarsaiya S. Binod P.. (2021). Bioengineered biochar as smart candidate for resource recovery toward circular bio-economy: a review. Bioengineered 12, 10269–10301. doi: 10.1080/21655979.2021.1993536, PMID: 34709979 PMC8809956

[ref46] Liu S. Meng J. Jiang L. Yang X. Lan Y. Cheng X. Y.. (2017). Rice husk biochar impacts soil phosphorous availability, phosphatase activities and bacterial community characteristics in three different soil types. Appl. Soil Ecol. 116, 12–22. doi: 10.1016/j.apsoil.2017.03.020

[ref47] Liu S. W. Xu M. Tuo L. Li X. J. Hu L. Chen L.. (2016). *Phycicoccus endophyticus* sp. nov., an endophytic actinobacterium isolated from *Bruguiera gymnorhiza*. Int. J. Syst. Evol. Microbiol. 66, 1105–1111. doi: 10.1099/ijsem.0.000842, PMID: 26653143

[ref48] Lu P. N. Bainard L. D. Ma B. Liu J. H. (2020). Bio-fertilizer and rotten straw amendments alter the rhizosphere bacterial community and increase oat productivity in a saline-alkaline environment. Sci. Rep. 10:19896. doi: 10.1038/s41598-020-76978-3, PMID: 33199781 PMC7669890

[ref49] Ma C. Naidu R. Liu F. Lin C. Ming H. (2012). Influence of hybrid giant Napier grass on salt and nutrient distributions with depth in a saline soil. Biodegradation 23, 907–916. doi: 10.1007/s10532-012-9583-4, PMID: 22899179

[ref50] Milton M. Bisarya D. Kumar V. Singh A. K. Mehta C. M. (2020). Microbial fertilizers: their potential impact on environment sustainability and ecosystem services. Int J Chem Stud. 8, 2308–2315. doi: 10.22271/chemi.2020.v8.i6ag.11120

[ref51] Moraditochaee M. Azarpour E. Bozorgi H. R. (2014). Study effects of bio-fertilizers, nitrogen fertilizer and farmyard manure on yield and physiochemical properties of soil in lentil farming. Int. J. Biosci. 4, 41–48. doi: 10.12692/ijb/4.4.41-48

[ref9003] Naeem M. A. Khalid M. Aon M. Abbas G. Amjad M. Murtaza B.. (2017). Combined application of biochar with compost and fertilizer improves soil properties and grain yield of maize. J. Plant Nutr. doi: 10.1080/01904167.2017.1381734

[ref52] Nassar A. H. El-Tarabily K. A. Sivasithamparam K. (2003). Growth promotion of bean (*Phaseolus vulgaris* L.) by a polyamine-producing isolate of *Streptomyces griseoluteus*. Plant Growth Regul. 40, 97–106. doi: 10.1186/s12866-022-02492-3

[ref53] Nisar B. Rashid S. Majeed L. R. Pahalvi H. N. Kamili A. N. (2021). “Introduction to microbiota and biofertilizers” in Microbiota and biofertilizers, vol. 2 (Cham: Springer), 195–232.

[ref54] Obermeier M. M. Minarsch E. M. L. Durai Raj A. C. Rineau F. Schröder P. (2020). Changes of soil-rhizosphere microbiota after organic amendment application in a *Hordeum vulgare* L. short-term greenhouse experiment. Plant and Soil 455, 489–506. doi: 10.1007/s11104-020-04637-7

[ref55] Qadir M. Oster J. D. Schubert S. Noble A. D. Sahrawat K. L. (2007). Phytoremediation of sodic and saline-sodic soils. In Donald LS (ed). Adv. Agron. 96, 197–247. doi: 10.1016/S0065-2113(07)96006-X

[ref56] Qiao Y. F. Miao S. J. Zhong X. Zhao H. F. Pan S. Q. (2020). The greatest potential benefit of biochar return on bacterial community structure among three maize-straw products after eight-year field experiment in Mollisols. Appl Soil Eco. 147:103432. doi: 10.1016/j.apsoil.2019.103432

[ref57] Qiu X. W. Zhou G. X. Zhang J. B. Wang W. (2019). Microbial community responses to biochar addition when a green waste and manure mix are composted: a molecular ecological network analysis. Bioresour. Technol. 273, 666–671. doi: 10.1016/j.biortech.2018.12.001, PMID: 30528727

[ref58] Rajput V. D. Minkina T. Chen Y. N. Sushkova S. Chapligin V. A. Mandzhieva S. (2016). A review on salinity adaptation mechanism and characteristics of *Populus euphratica*, a boon for arid ecosystems. Acta Ecol. Sin. 36, 497–503. doi: 10.1016/j.chnaes.2016.08.001

[ref59] Rani N. Kaur G. Kaur S. Upadhyay S. K. Tripathi M. (2022). Development of Zn biofertilizer microbeads encapsulating *Enterobacter ludwigii*-PS10 mediated alginate, starch, poultry waste and its efficacy in *Solanum lycopersicum* growth enhancement. Int. J. Biol. Macromol. 240:124381. doi: 10.1016/j.ijbiomac.2023.124381 37044325

[ref60] Ren C. G. Bai Y. J. Kong C. C. Bian B. Xie Z. H. (2016). Synergistic interactions between salt-tolerant rhizobia and arbuscular mycorrhizal fungi on salinity tolerance of *Sesbania cannabina* plants. J. Plant Growth Regul. 35, 1098–1107. doi: 10.1007/s00344-016-9607-0

[ref61] Ren H. Huang B. Fernández-García V. Miesel J. Yan L. Lv C. Q. (2020). Biochar and rhizobacteria amendments improve several soil properties and bacterial diversity. Microorganisms 8:502. doi: 10.3390/microorganisms8040502, PMID: 32244714 PMC7232174

[ref62] Ren C. G. Kong C. C. Xie Z. H. (2018). Role of abscisic acid in strigolactone-induced salt stress tolerance in arbuscular mycorrhizal *Sesbania cannabina* seedlings. BMC Plant Biol. 18, 74–10. doi: 10.1186/s12870-018-1292-7, PMID: 29724168 PMC5934815

[ref63] Saifullah Dahlawi S. Naeem A. Rangel Z. Naidu R. (2018). Biochar application for the remediation of salt-affected soils: challenges and opportunities. Sci. Total Environ. 625, 320–335. doi: 10.1016/j.scitotenv.2017.12.257, PMID: 29289780

[ref64] Saleh A. M. Madany M. (2015). Coumarin pretreatment alleviates salinity stress in wheat seedlings. Plant Physiol. Biochem. 88, 27–35. doi: 10.1016/j.plaphy.2015.01.005, PMID: 25634803

[ref65] Sarfraz R. Yang W. H. Wang S. S. Zhou B. Q. Xing S. H. (2020). Short term effects of biochar with different particle sizes on phosphorous availability and microbial communities. Chemosphere 256:126862. doi: 10.1016/j.chemosphere.2020.126862, PMID: 32442795

[ref66] Sun J. H. Yang L. Wei J. Quan J. N. Yang X. T. (2020). The responses of soil bacterial communities and enzyme activities to the edaphic properties of coal mining areas in Central China. PLoS One 15:e0231198. doi: 10.1371/journal.pone.0231198, PMID: 32343698 PMC7188301

[ref67] Tao C. Y. Li R. Xiong W. Shen Z. Z. Liu S. S. Wang B. B.. (2020). Bio-organic fertilizers stimulate indigenous soil Pseudomonas populations to enhance plant disease suppression. Microbiome 8, 137–114. doi: 10.1186/s40168-020-00892-z, PMID: 32962766 PMC7510105

[ref68] Upadhyay S. K. Chauhan P. K. (2022). Optimization of eco-friendly amendments as sustainable asset for salt-tolerant plant growth-promoting bacteria mediated maize (*zea mays* L.) plant growth, Na uptake reduction and saline soil restoration. Environ. Res. 211, 211:113081. doi: 10.1016/j.envres.2022.113081, PMID: 35304115

[ref69] Upadhyay S. K. Rajput V. D. Kumari A. Espinosa-Saiz D. Menendez E. Minkina T.. (2022a). Plant growth-promoting rhizobacteria: a potential bio-asset for restoration of degraded soil and crop productivity with sustainable emerging techniques. Environ. Geochem. Health. 1–24. doi: 10.1007/s10653-022-01433-3, PMID: 36413266

[ref70] Upadhyay S. K. Srivastava A. K. Rajput V. D. Chauhan P. K. Bhojiya A. A. Jain D.. (2022b). Root exudates: mechanistic insight of plant growth promoting rhizobacteria for sustainable cropproduction. Front. Microbiol. 13:916488. doi: 10.3389/fmicb.2022.916488, PMID: 35910633 PMC9329127

[ref71] Wang J. H. Wang X. L. Wang K. Ren Z. X. Wang M. L. (2021). Effects of replacing chemical fertilizers with bio-organic fertilizers on microenvironment of wheat rhizosphere soil. Acta Agric Boreali Sin. 36, 155–162. doi: 10.7668/hbnxb.20191894

[ref72] Wu S. L. Xue S. Iqbal Y. Xing H. C. Jie Y. C. (2021). Seasonal nutrient cycling and enrichment of nutrient-related soil microbes aid in the adaptation of ramie (*Boehmeria nivea* L.) to nutrient-deficient conditions. Front. Plant Sci. 12:644904. doi: 10.3389/fpls.2021.644904, PMID: 33868344 PMC8044408

[ref73] Xiao L. Meng F. D. (2020). Evaluating the effect of biochar on salt leaching and nutrient retention of Yellow River Delta soil. Soil Use Manage. 36, 740–750. doi: 10.1111/sum.12638

[ref74] Xie T. Reddy K. R. Wang C. Yargicoglu E. Spokas K. (2015). Characteristics and applications of biochar for environmental remediation: a review. Crit. Rev. Environ. Sci. Technol. 45, 939–969. doi: 10.1080/10643389.2014.924180

[ref75] Xu D. D. Ling J. F. Qiao F. Xi P. G. Zeng Y. N. Zhang J. F.. (2022). Organic mulch can suppress litchi downy blight through modification of soil microbial community structure and functional potentials. BMC Microbiol. 22, 155–112. doi: 10.1186/s12866-022-02492-3, PMID: 35689202 PMC9188084

[ref76] Xu L. X. Yi M. Yi H. L. Guo E. Zhang A. Y. (2018). Manure and mineral fertilization change enzyme activity and bacterial community in millet rhizosphere soils. World J. Microbiol. Biotechnol. 34, 8–13. doi: 10.1007/s11274-017-2394-3, PMID: 29236189

[ref77] Yang M. Yang R. Y. Li Y. N. Pan Y. H. Zhang Z. H. (2021). Effects of different biomass materials as a salt-isolation layer on water and salt migration in coastal saline soil. Peer J. 9:e11766. doi: 10.7717/peerj.11766, PMID: 34277156 PMC8272462

[ref78] Yao Q. Liu J. J. Yu Z. H. Li Y. S. Jin J. Liu X. B.. (2017). Three years of biochar amendment alters soil physiochemical properties and fungal community composition in a black soil of Northeast China. Soil Biol. Biochem. 110, 56–67. doi: 10.1016/j.soilbio.2017.03.005

[ref79] Yu Y. Y. Li S. M. Qiu J. P. Li J. G. Luo Y. M. Guo J. H. (2019). Combination of agricultural waste compost and biofertilizer improves yield and enhances the sustainability of a pepper field. J. Plant Nutr. Soil Sci. 182, 560–569. doi: 10.1002/jpln.201800223

[ref80] Yuan J. H. Xu R. K. Qian W. Wang R. H. (2011). Comparison of the ameliorating effects on an acidic ultisol between four crop straws and their biochars. J. Soil. Sediment. 11, 741–750. doi: 10.1007/s11368-011-0365-0

[ref81] Yue Y. Guo W. N. Lin Q. M. Li G. T. Zhao X. R. (2016). Improving salt leaching in a simulated saline soil column by three biochars derived from rice straw (*Oryza sativa* L.), sunflower straw (*Helianthus annuus*), and cow manure. J. Soil Water Conserv. 71, 467–475. doi: 10.2489/jswc.71.6.467

[ref9004] Zhang M. Jin B. J. Bi Q. F. Li K. J., and Zhu Y. G. (2021). Variations of earthworm gut bacterial community composition and metabolic functions in coastal upland soil along a 700-year reclamation chronosequence. Science of The Total Environment, 804, 149994. doi: 10.1016/j.scitotenv.2021.149994 34798714

[ref82] Zhang D. J. Li J. J. Huang Y. M. Gao S. Zhang J. (2022). Root-soil facilitation in mixed Eucalyptus grandis plantations including nitrogen-fixing species. Forest Ecol Manag. 516, 120215. doi: 10.1186/s12866-022-02492-3

[ref83] Zhang H. Q. Wu J. Han S. Xi Y. D. Li Y. J. Liang G. Y. (2022). Effects of four annual rotation patterns on soil microbial community. Chin Agric Sci Bull. 38, 73–80.

[ref84] Zhao S. Chen X. Deng S. P. Dong X. N. Song A. P. Yao J. J. (2016). The effects of fungicide, soil fumigant, bio-organic fertilizer and their combined application on chrysanthemum *Fusarium* wilt controlling, soil enzyme activities and microbial properties. Molecules 21:526. doi: 10.3390/molecules21040526, PMID: 27110753 PMC6273536

[ref85] Zhao Y. G. Wang S. J. Li Y. Liu J. Zhuo Y. Q. Wang J.. (2018). Long-term performance of flue gas desulfurization gypsum in a large-scale application in a saline-alkali wasteland in Northwest China. Agric. Ecosyst. Environ. 261, 115–124. doi: 10.1016/j.agee.2018.01.009

[ref86] Zheng Y. Liang J. Zhao D. L. Meng C. Zhang C. S. (2020). The root nodule microbiome of cultivated and wild halophytic legumes showed similar diversity but distinct community structure in yellow river delta saline soils. Microorganisms 8:207. doi: 10.3390/microorganisms8020207, PMID: 32028717 PMC7074777

[ref87] Zheng H. Wang X. Chen L. Wang Z. Y. Xia Y. Zhang Y. P.. (2018). Enhanced growth of halophyte plants in biochar-amended coastal soil: roles of nutrient availability and rhizosphere microbial modulation. Plant Cell Environ. 41, 517–532. doi: 10.1111/pce.12944, PMID: 28349546

[ref88] Zhou G. Xu X. Qiu X. Zhang J. (2018). Biochar influences the succession of microbial communities and the metabolic functions during rice straw composting with pig manure. Bioresour. Technol. 272, 10–18. doi: 10.1016/j.biortech.2018.09.135, PMID: 30292912

[ref9006] Zhu Y. Zhong M. Li W. Qiu Y. Wang H. Lv X. (2022). Cotton straw biochar and bacillus compound biofertilizer decreased cd migration in alkaline soil: insights from relationship between soil key metabolites and key bacteria. Ecotox. Environ. Safety. 232, 113293. doi: 10.1016/j.ecoenv.2022.113293 35158279

