# Supplementary material for: Effect of biochar and bioorganic fertilizer on the microbial diversity in the rhizosphere soil of Sesbania cannabina in saline-alkaline soil
Source: Front Microbiol. 2023 Jun 29;14:1190716. doi: 10.3389/fmicb.2023.1190716 (PMC10339320; doi:10.3389/fmicb.2023.1190716)
Supplement: Supplementary file 1 [file Data_Sheet_1.docx]

Supplementary Material

**Table S1 PCR details**

|  | experiment details |
| --- | --- |
| **soil physicochemical properties** | Available phosphorous (AP) was extracted from soil samples using 0.5 M NaHCO_3_, and measured using a segmented continuous flow analyzer (Quaatro, Bran+Luebbe, Germany). Alkaline nitrogen (AN) and total nitrogen (TN) contents of soil were determined using an elemental analyzer (FLASH-2000; Thermo Scientific, USA). Available potassium (AK) was extracted using 1 M ammonium acetate (pH 7.0), and quantified using an Atomic Absorption Spectrophotometer AA4590. Soil OM was quantitated using the potassium dichromate oxidation method, and soil OC was determined using the SSM-5000A (Shimadzu, Japan) carbon analyzer. |
| PCR mixtures | The PCR mixtures contained 4 μL of 5x TransStartFastPfu buffer, 2 μL of 2.5 mM dNTPs, 0.8 μL of each primer (5 μM each), 0.4 μL of *TransStart*FastPfu DNA Polymerase, 10 ng template DNA, adding ddH_2_O to a final volume of 20 μL. All reactions were performed in triplicate. |
| PCR cycling conditions | PCR cycling conditions included an initial denaturation at 95°C for 3 min, 27 cycles of denaturing at 95°C for 30 s, annealing at 55°C for 30 s, and extension at 72°C for 45 s, followed by a single extension at 72°C for 10 min and a continued hold at 4°C. |
| Splicing and quality control | Raw sequence reads were demultiplexed, quality-filtered by fastp version 0.20.0, and merged by FLASH version 1.2.7. Operational taxonomic units (OTUs), with a 97% similarity cut-off, were clustered using UPARSE v.7.1, and chimeric sequences were identified and removed. The taxonomy of each OTU representative sequence was analyzed by RDP Classifier v.2.2 against the 16S rRNA database using a confidence threshold of 0.7. |

**
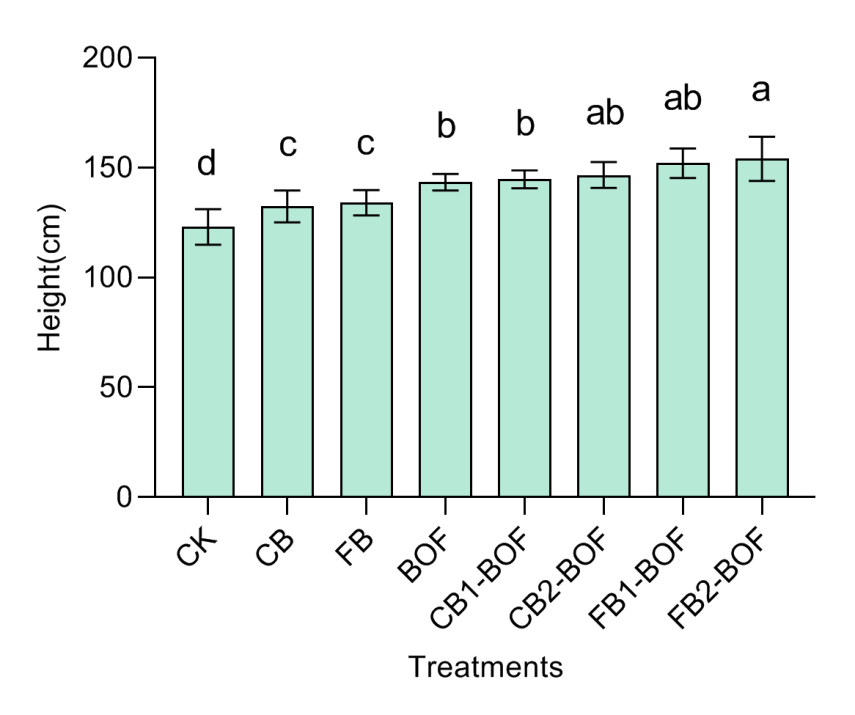
**

**Fig S1 The plant height of *Sesbania cannabina***

*Note: Data represent mean ± standard deviation (SD) of three biological replicates. Different lowercase letters indicate significant differences (P < 0.05).*


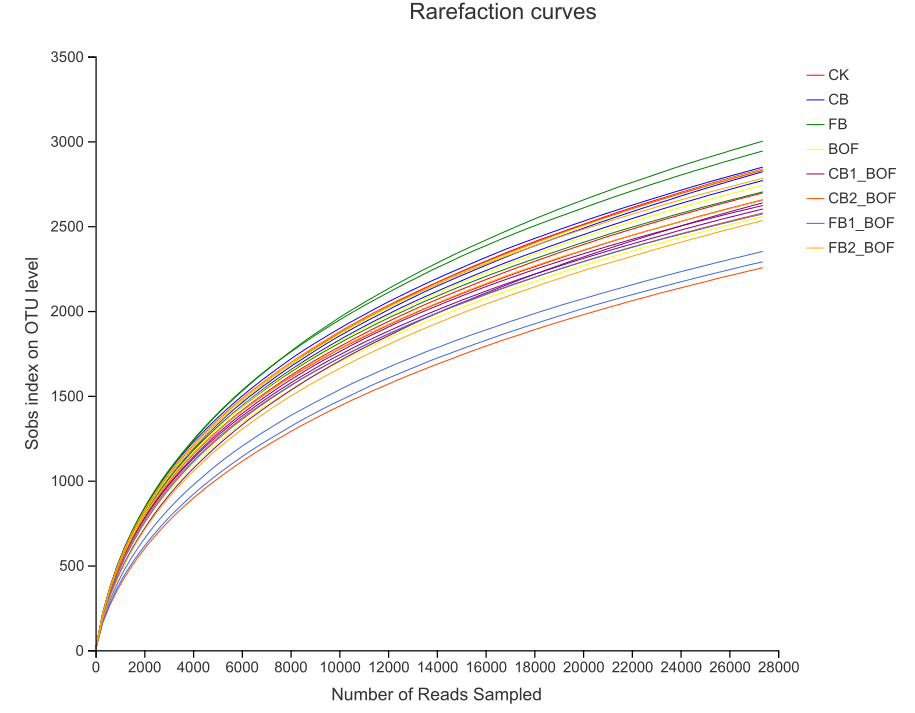


**Fig S2 Rarefaction curves depicting the number of OTUs with 97% similarity identified from different samples**

*Note: CK: soil without amendment; CB: soil amended with 10 mesh biochar; FB: soil amended with 30 mesh biochar; BOF: soil amended with BOF; CB1_BOF (100 t ha^−1^ 10 mesh biochar + BOF), CB2_BOF (150 t ha^−1^ 10 mesh biochar + BOF), FB1_BOF (100 t ha^−1^ 30 mesh biochar + BOF), FB2_BOF (150 t ha^−1^ 30 mesh biochar + BOF); Error bars represent the standard error of mean (n = 3).*

**Table S2 Operational taxonomic unit richness and diversity indices of different samples**

|  | sobs | shannon | simpson | ace | chao | coverage |
| --- | --- | --- | --- | --- | --- | --- |
| CK | 2729.33±77.95ab | 6.54±0.02ab | 0.005±0.0003c | 4184.69±237.55a | 3939.58±58.84ab | 0.963±0.001ab |
| CB | 2812.67±32.79ab | 6.61±0.05a | 0.004±0.0002c | 4629.88±395.76a | 4213.09±128.08a | 0.961±0.001b |
| FB | 2882±129.46a | 6.64±0.03a | 0.004±0.0004c | 4137±228.56a | 4088.43±181.14a | 0.961±0.002b |
| BOF | 2711±115.46ab | 6.5±0.08ab | 0.005±0.001c | 4438.26±448.94a | 4055.82±210.52a | 0.962±0.002b |
| CB1-BOF | 2404.33±121.15c | 5.92±0.24c | 0.018±0.005a | 3915.04±201.85a | 3523.11±103.47b | 0.967±0.0003a |
| CB2-BOF | 2656±101.68abc | 6.42±0.21ab | 0.007±0.003bc | 4218.91±228.55a | 3864.34±4.64ab | 0.964±0.0001ab |
| FB1-BOF | 2620±14.76abc | 6.37±0.14ab | 0.007±0.002bc | 4246.36±425.03a | 3886.73±148.95ab | 0.964±0.002ab |
| FB2-BOF | 2554.33±235.34bc | 6.18±0.38bc | 0.011±0.005b | 4188.01±433.58a | 3787.68±311.13ab | 0.964±0.002ab |

*Data represent mean ± standard deviation (SD) of three biological replicates. Different lowercase letters within a column indicate significant differences (P < 0.05).*

**Table S3 The table of top relative abundance among treatments**

|  | CK | CB | FB | BOF | CB1_BOF | CB2_BOF | FB1_BOF | FB2_BOF |
| --- | --- | --- | --- | --- | --- | --- | --- | --- |
| phylum | Actinobacteriota | Actinobacteriota | Proteobacteria | Actinobacteriota | Proteobacteria | Actinobacteriota | Actinobacteriota | Proteobacteria |
| class | Alphaproteobacteria | Alphaproteobacteria | Alphaproteobacteria | Alphaproteobacteria | Alphaproteobacteria | Actinobacteria | Actinobacteria | Alphaproteobacteria |
| order | Tistrellales | Vicinamibacterales | Tistrellales | Vicinamibacterales | Tistrellales | Micromonosporales | Micromonosporales | Tistrellales |
| family | Geminicoccaceae | Geminicoccaceae | Geminicoccaceae | Geminicoccaceae | Geminicoccaceae | Micromonosporaceae | Micromonosporaceae | Geminicoccaceae |
| genus | norank_f__Geminicoccaceae | norank_f__Geminicoccaceae | norank_f__Geminicoccaceae | norank_f__Geminicoccaceae | norank_f__Geminicoccaceae | Micromonospora | Micromonospora | norank_f__Geminicoccaceae |

**Table S4 Correlation network analysis of microbial communities**

|  | | CB-FB | CB1_BOF-FB1_BO F | CB2_BOF-FB2_BOF |
| --- | --- | --- | --- | --- |
| Node numbers | Soil parameter | 5 | 6 | 6 |
|  | Bacteria | 15 | 7 | 21 |
| Edge numbers | | 21 | 10 | 30 |
| Node average degree | | 2 | 1.5 | 2.1 |
| Positive edges | | 16 | 6 | 27 |
| Negative edges | | 5 | 4 | 3 |

*The correlation network indices were calculated based on the top 30 genera. The average number of connections per node in the network, that is, the node connectivity.*
